# Imaging of Lithium Ion Release from Individual Cathode Particles Shows Evidence for Diversity of Intraparticle Contacts

**DOI:** 10.1021/acs.chemmater.5c00906

**Published:** 2025-07-17

**Authors:** Andrew C. Cavell, Evan T. Jensen, Benjamin A. Brewster, Mengcheng Wu, Mackinsey A. Smith, Michael S. Mattei, Rachel Czerwinski, Lucy C. Kneeley, Michael S. Foy, Aisley F. Fleming, Elisa T. Harrison, Sabrina L. Peczonczyk, Alvaro G. Masias, Randall H. Goldsmith

**Affiliations:** † Department of Chemistry, 5228 University of Wisconsin-Madison, 1101 University Avenue, Madison, Wisconsin 53705, United States; ‡ 1931 Ford Motor Company, 2101 Village Road, Dearborn, Michigan 48121, United States

## Abstract

The ability to observe nanoscale transport of lithium ions in battery functional components with high spatial and temporal resolution can offer a powerful source of mechanistic insight. While existing methods can provide dynamics for single nanocrystals of cathode materials, the behavior of macroscopic battery systems cannot be extrapolated from this single-crystal limit. Key elements of these systems’ complexity, such as those arising from particle–particle contacts, only emerge after the transition to the mesoscopic length scale. In this work we demonstrate how fluorescence imaging can be used to watch the electrochemically initiated release of lithium ions from mesoscopic lithium cobalt oxide cathode particles and to quantify the quantity of lithium released as a function of time. The particle-to-particle diversity of release kinetics observed with this method is remarkable. Additionally, correlative scanning electron microscopy of the particles highlights links between certain structural features and lithium storage capacity, while also revealing unexpected weaker correlations between structural motifs and the time scales of the dynamics. Importantly, the complexity of these dynamics provides evidence for a wide distribution of intraparticle contact resistances between nanocrystalline domains, a distribution that was previously hypothesized to be present but not yet directly observed. Quantification of the intraparticle lithium diffusion constant shows the robustness of the approach. This ability to quantify lithium ion release behavior from mesoscopic particles therefore offers a powerful path for obtaining chemical insight on this important length scale in between single nanocrystals and macroscopic devices.

## Graphical abstract

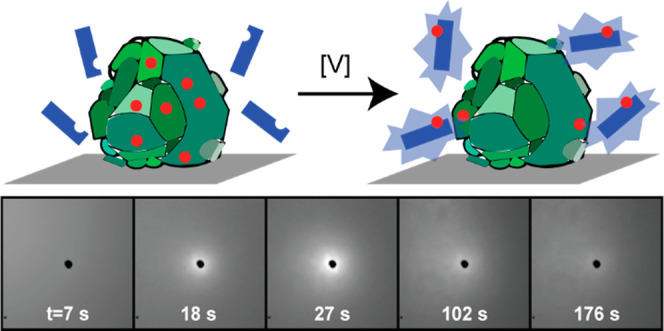

## Introduction

Developing better energy storage materials for increased density, capacity, and safety is essential to support the increasing demand in and shifting modes of energy utilization on a global scale.^1^ Lithium-ion batteries in particular constitute a huge share of these initiatives, owing to their high energy densities and low cost.^2,3^ While some alternatives are being developed, lithium-ion batteries are still the most accessible and widely deployed secondary cells, and so increasing their performance and safety is of particular importance.^4^

Battery electrodes are inherently complex multiscale structures with chemical processes occurring at the nano-, meso-, and macroscopic scales. Consequently, many diagnostic techniques exist to evaluate performance and elucidate mechanism at these varied length scales. While a variety of electrochemical and spectroscopic whole-device diagnostic tools are in use,^5–13^ diagnostic tools at the nanoscale, including techniques that offer spatial resolution with the potential for single-particle resolution are growing in importance.^14–28^ Some of these methods provide post-mortem analyzes of particles, following the movement of lithium by mapping lithium density in individual particles or whole electrodes disassembled after cycling.^29–31^ In one example, Sugar et al. used high resolution energy-filtered TEM maps of individual cathode particles to show that most monocrystalline particles are either fully lithiated or fully delithiated.^31^ Some measurements combine this spatial resolution with the ability to follow lithium migration in real time, providing additional insight into the dynamics of these processes. X-ray absorption has been used to monitor lithiation and rate of transport from single crystalline particles of LiFePO_4_ and from solid lithium electrodes *in operando*.^32–34^ Similarly, interferometric scattering (iSCAT) has been used to image lithiation and delithiation in single crystalline cathode particles of LiCoO_2_ (LCO)^35^ and anode particles of Nb_14_W_3_O_44_ ^36^ by monitoring changes in refractive index. Surface plasmon resonance (SPR) measurements can monitor refractive index changes as a result of lithiation of single LCO particles, extracting indirect single-particle electrochemical traces.^37^ Single-particle studies using TEM allow observation of lithiation kinetics at crystalline Si nanoparticles and at single FeF_2_ particles.^19,20^

Though the ability to monitor chemical behavior across multiple levels of organization could enable greater understanding of the behavior of electrode materials, diagnostic tools that allow such multiscale monitoring are largely unknown. Moving through the hierarchy (Figure 1) from the single-crystal level (with a length scale of hundreds of nm) to a whole electrode, up to an entire cell (>1 mm) leads to increasing numbers of interactions and ever higher levels of complexity. Many of the nanoscale imaging measurements mentioned above examine only single nanocrystals,^32–36^ and do not possess the required depth of field^35^ to examine assemblies of particles. Often these methods must rely on measuring only a single crystal in order to obtain clean, interpretable data. Though these studies yield valuable microscopic insights, one cannot expect to accurately or fully describe the macroscopic system by extrapolating the behavior of a single crystal to the scale of a full electrode. Indeed, researchers developing computational methods to describe battery dynamics have already recognized the need for multiscale modeling beyond the extremes of a single particle or full electrode,^38–43^ and experimental efforts have highlighted the importance of short-range electronic contacts.^44^ However, most experimental techniques operate at these very extremes. When intermediate scales are included in the computational models, their parameters are typically estimated by fitting to bulk data,^41^ resulting in underdetermination of parameters. Importantly, being able to experimentally examine mesoscale interactions such as those among small numbers of crystals before they are placed into the densely packed environment of a full manufactured electrode may reveal insights into the behavior of these materials that are absent in the single-crystal case, but impossible to resolve in the full electrode. Information gained between the extremes could unlock a greater understanding of the observed macroscale phenomena at the electrode by connecting intraparticle and interparticle dynamics to the performance of the whole cell.

**Figure 1:**
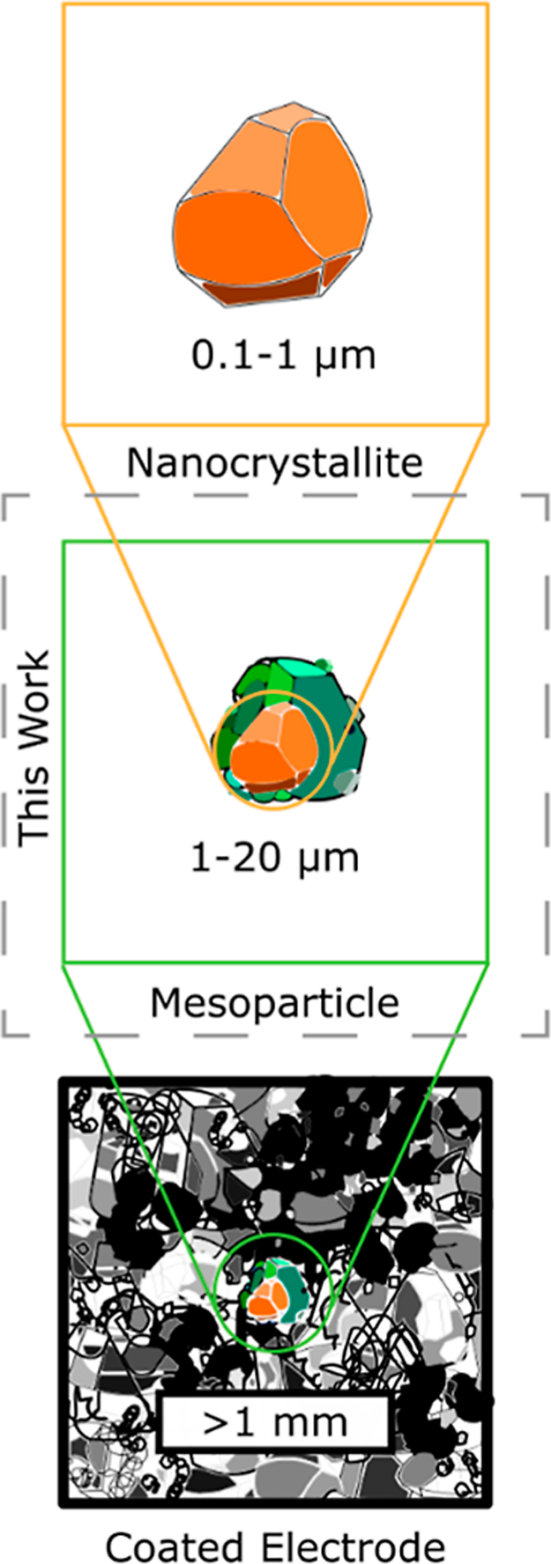
Different length scales accessed by battery characterization techniques. Rather than focus on the extremes of individual crystals of LCO or of fully fabricated electrodes composed of many particles packed together, this work examines behavior at the mesoparticle level, wherein the particles are micron-scale entities composed of many nanocrystallites.

Here, we present a method for monitoring the dynamics of lithium-ion release from mesoscale (Figure 1, middle panel) cathode particles using fluorescence microscopy and demonstrate its ability to gain insight into the underlying mechanism. Our method employs the lithium-ion sensitive fluorescent dye 2-(2-hydroxyphenyl)-naphthoxazole (HPNO), Figure 2a, which we have previously shown fluoresces upon binding to lithium ions in solution, allowing for fluorescence imaging of local changes in lithium-ion concentration.^14^ Notably, while most examples of *in situ* measurements of lithiation or delithiation focus on the changing morphology of the solid matrix, very few directly monitor transport of ions from the electrode into the electrolyte solution outside of the particle, a fundamental step in battery charging. Here, lithium discharge from cathode particles is monitored *in situ*. A major strength of the technique is the high temporal and spatial resolution afforded by fluorescence microscopy combined with specificity for Li^+^ and the ability to probe complex 3D polycrystalline mesoparticles. A deconvolution procedure can then be used to extract release traces from the fluorescence data, moles of released lithium can be determined, and correlations between lithium release and particle morphology as measured by SEM can be explored. However, this technique is limited by the dynamic range over which the fluorescent sensor can be quantitatively correlated with a lithium ion concentration, and thus the overall lithium concentration in the electrolyte must be kept low.^14^ Therefore, while this method allows for robust quantification of lithium diffusion constants in solution at individual polycrystalline particles, as shown below, the concentration limitation likely prevents application to electrodes produced from slurries or commercial electrolyte solutions that contain high concentrations of Li^+^. This limit also prevents repeated sampling over multiple charge and discharge cycles, though the method is still valuable for measurements of lithium release early in the charging process.

**Figure 2:**
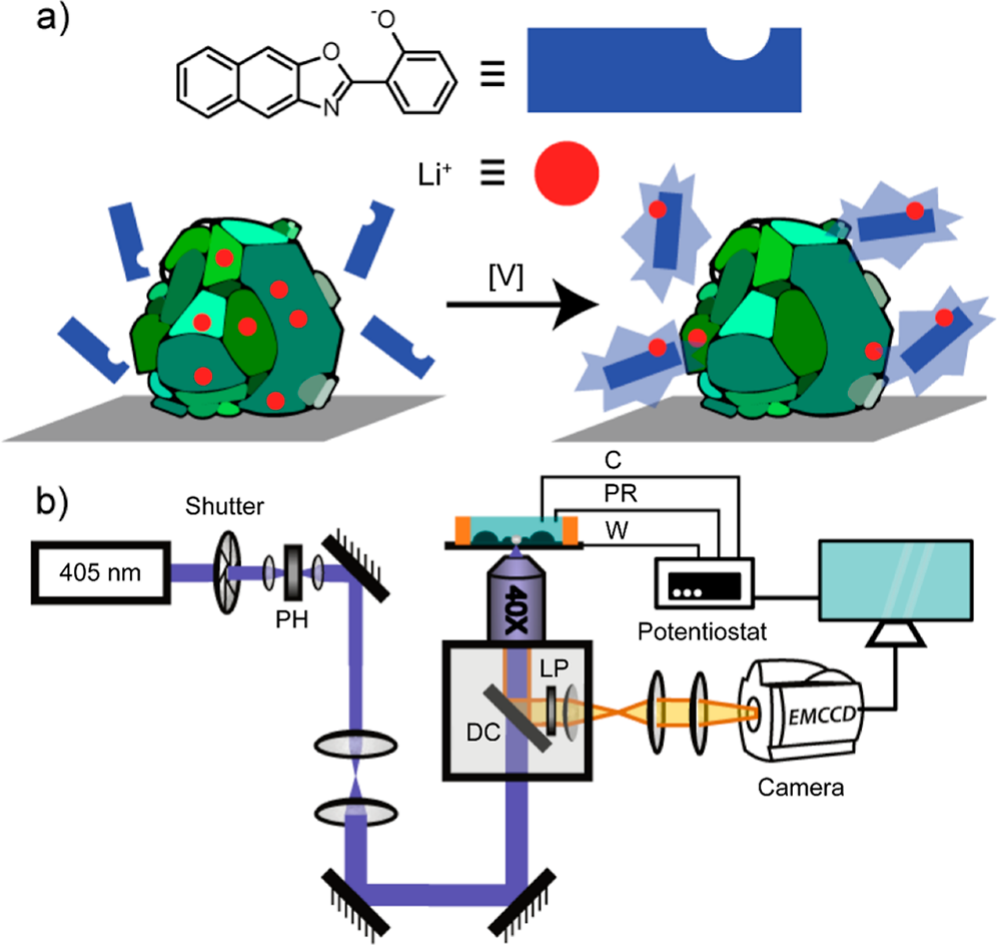
(a) The HPNO fluorophore fluoresces intensely upon binding to lithium ions when excited at 405 nm. Particles of LCO release Li^+^ into solution when an oxidizing voltage is applied, leading to the observed fluorescent signal. (b) Diagram of fluorescence microscopy and electrochemical setup used in this work. PH: Pinhole. DC: Dichroic. LP: Long-pass filter. W: Working electrode. C: Platinum counter electrode. PR: Silver wire pseudo-reference electrode.

We demonstrate this method on particles of LiCoO_2_ (LCO), chosen due to its popularity as a commercial cathode material and use in a significant share of lithium-ion batteries in portable electronics.^45–47^ The LCO-based cathodes used in these cells are inherently multiscale, with polycrystalline mesoparticles composed of multiple LCO nanocrystallites clumped together in close contact with one another, thus allowing an opportunity to investigate the behavior of small collections of nanocrystallites of LCO, and therefore to bridge the gap between studies of single particles and those of manufactured electrodes (Figure 1). Further, this method is generalizable to the study of a wide variety of lithium-ion battery materials. Our method joins a small but growing number of other methods capable of resolving fundamental electrochemical events using turn-on fluorescence techniques.^48–54^

## Experimental Methods

### Preparation of LiCoO_2_ Samples

Samples of LiCoO_2_ particles (Electrodes and More, BLCOP-25) were prepared on transparent, conductive coverslips for use in all experiments. The coverslips used were indium tin oxide (ITO) on glass (Delta Technologies CB-50IN-01015, 120–160 nm ITO on 0.15 mm glass, *R*_s_ = 5–15 ohms). These coverslips were scored with an asymmetric pattern to facilitate location of individual particles for correlative fluorescence microscopy and scanning electron microscopy (SEM). The samples were prepared by suspending LCO powder in 2-propanol (Fisher Chemical, HPLC grade) at a concentration of 0.25 mg/mL, which was then deposited by spin-coating on the ITO coverslips (80 μL of solution, 4000 rpm, 10 s). The prepared coverslips, shown in Figure S9, were used for SEM and electrochemistry/fluorescence experiments. Additional details are provided in the Supporting Information.

### Cell Construction and Electrochemistry

All experiments were conducted in bulk electrolysis (controlled-potential coulometry) mode on a CH Instruments 620E potentiostat unless otherwise noted. The cells were constructed atop the prepared LCO/ITO coverslip samples by adhering a hybridization chamber (SecureSeal, Grace Bio-Laboratories, 8 mm exposed diameter) directly to the coverslip itself (see Supporting Information for details). Copper tape was used to contact the ITO surface, and a Pt wire counter electrode was inserted through the top of the chamber. With the LCO/ITO acting as a working electrode and an additional Ag wire as a pseudoreference electrode, this configuration affords a 3-electrode cell, Figure 2b, for use in electrochemical measurements. The cell was filled with a solution of HPNO fluorophore (3.8 mM), synthesized as previously reported by our lab,^14^ dissolved in propylene carbonate (MilliporeSigma, anhydrous) with 4% v/v triethylamine (MilliporeSigma) as solvent. The 4% triethylamine keeps the HPNO molecule deprotonated in order to allow it to bind to lithium ions. The solution also contained 0.1 M TBAPF_6_ (MilliporeSigma) as an electrolyte. All solvents were used as received.

In each experiment, 2 s are allowed to elapse before voltage is applied to collect a baseline current and background fluorescence intensity. The potentiostat then applies 1 V (vs the Ag reference, approximately equal to 3.3–3.4 V vs Li/Li^+^, see Supporting Information) to the entirety of the ITO working electrode and holds that potential for the duration of the experiment. The potential between the working and counter electrode is independently recorded from the potentiostat’s analog outputs using a data acquisition card (National Instruments DAQ) to confirm synchronization. The typical rise time of the 620E potentiostat is <1 μs and was verified independently for each experiment to be ≪10 ms (limited by the rate at which samples were taken from the NI-DAQ). The rise of the 1 V square wave is instantaneous on the time scale of the imaging. Galvanostatic (constant voltage) conditions can also be applied (see Figure S33). In general, the current from a single particle, or from an entire coverslip sparsely covered with particles, is too low to measure, but current can be measured from a more heavily loaded sample during discharge (see Figure S29). LCO particles without the HPNO sensor show negligible fluorescence intensity changes (see Supporting Information). Additional details are provided in the Supporting Information.

### Optical System

A custom widefield fluorescence microscope was used for imaging of the LCO particles, composed of an inverted microscope (Nikon TE-2000U) with a 405 nm diode laser (Cobolt MLD, 120 mW, 0.2 W/cm^2^) providing a beam that was spatially filtered through a 50 μm pinhole and delivered to the sample through a 40× Nikon objective (0.65 NA, air), Figure 2b. A series of telescopes resulted in an excitation region approximately 250 μm in diameter in the sample plane. A 405 nm dichroic (Semrock Di02-R405-25 × 36) was used to separate the emission from the excitation light. The emission channel was further filtered with a long pass filter (Semrock BLP01-405R-25) to completely remove the excitation photons from the measured signal. An EMCCD camera (Andor Ixon 897) was configured to take fluorescence videos immediately upon initiation of the experiment by the potentiostat with synchronization and data acquisition automated using a custom LabVIEW interface. Unless stated otherwise, each experiment used a 100 ms exposure time. The CCD was cooled to −80C during data acquisition. Frames were taken consecutively for up to 6900 repetitions to acquire up to 15 min-long videos for each experiment at a frame rate of 7.56 frames per second. Workup of the fluorescence videos to produce standardized traces was achieved through custom analysis software written in MATLAB. Additional details are provided in the Supporting Information.

### Diffusion Simulations

Finite element numerical simulations of the solution-phase lithium-ion diffusion from single particle sources were produced using COMSOL Multiphysics. The simulation itself includes a hemispherical concentrated region of infinitely small particles representing the lithium ions, a simple model that is representative of the small LCO particle in its lithiated state resting on a coverslip. The source region is defined to be 2.5 μm in radius (the size of a typical LCO particle) and is placed on a surface impermeable to diffusion. This simplified model does not account for the volume occupied by the particle itself or the irregular shape of the particles seen in experiments, but these impacts on the simulated diffusion traces are expected to be small, owing to the relatively large size of the ROI used for integration. After time point 0, the ions are allowed to diffuse freely, in three dimensions, in solution with a diffusion constant *D* = 1 × 10^–10^ m^2^/s. This value was extracted from experimental trajectories by imposing a constraint that *D* values obtained at increasingly large displacements from the center position must be the same (see Supporting Information). This value is notably close to values determined from NMR measurements of lithium ion diffusion in solutions of LiPF_6_ in propylene carbonate (*D* = 0.69 × 10^–10^ m^2^/s).^55^ In addition, in previous fluorescence microscopy experiments using HPNO to track lithium ions a diffusion constant of *D* = 2.11 × 10^–10^ m^2^/s was found, albeit with LiCl salt and the inclusion of a poly­(ethylene oxide) (PEO) supporting electrolyte.^14^ Thus, the above simulated value is supported by literature and consistent with other recent experimental investigation.

The concentration of particles inside a cylindrical volume of the same radius as the experimental region of interest (ROI) is plotted against time to yield the diffusion function. Modeling this way replicates the capture of fluorescence in a two-dimensional image that is a projection of the full 3D cylinder, as with the images used in later image analysis. The number of ions inside each cylinder is directly proportional to the fluorescence intensity inside a ROI at any given time.^14^ Additional details are provided in the Supporting Information.

## Results and Discussion

### Electrochemistry and *In Situ* Fluorescence Measurements

Electrochemical cells were constructed using indium tin oxide (ITO) coated glass coverslips which have been sparsely coated with lithium cobalt oxide (LCO) cathode particles. This transparent conductive ITO coated glass is chosen to allow an inverted microscope to be used for widefield fluorescence imaging of electrochemically initiated dynamics.^53^ Imaging through the bottom surface to directly observe the region around the LCO particles avoids losses and imaging distortions caused by imaging through the electrolyte solution and makes for simple construction of the 3-electrode electrochemical cell. The electrolyte solution contains a fluorescent tracer dye previously reported by our group, 2-(2-hydroxyphenyl)-naphthoxazole (HPNO),^14^ which shows a marked increase in fluorescence intensity upon binding to any freely diffusing lithium ions. As the particles of LCO release Li^+^ into the surrounding electrolyte solution during charging, the profluorophore HPNO binds to those lithium ions, leading to an increase in fluorescence around the particle that is easily observable in fluorescence imaging. This working principle is illustrated in Figure 2a and more comprehensive mechanistic detail has been previously presented.^14^ At high concentration, HPNO molecules are expected to bind to lithium ions in a 2:1 stoichiometry.^14^ Notably, the profluorophore has good selectivity for Li^+^ over other ions, reducing any potential interference in the fluorescent signal that could arise from contamination or trace transition metal dissolution.^14^ By using fluorescence imaging, high spatial and temporal resolution can be achieved, with the fluorescence intensity at any point in the field of view directly proportional to the local concentration of lithium at that point in space and time.^14^ In this work we only sought to study the cathode material (LCO), and so to simplify the experiment and analysis, a simple platinum counter electrode, instead of an ion intercalating anode that would be present in a battery, is present in our sample cell.

A typical fluorescence image containing an LCO particle is shown in Figure 3a. The potential applied to the electrode acts to oxidize the lithium cobalt oxide cathode material, causing deintercalation of lithium ions and their subsequent release into the surrounding solution, a fundamental microscopic process in battery charging. Upon this release, the lithium ions bind to HPNO in solution, and a plume of fluorescence is observed around each particle in the field of view that can be quantified to reveal temporal dynamics of lithium ion release. The full video of the particle in Figure 3a is available in the Supporting Information. The observed fluorescence is typically radially symmetric around the active particles. The average intensity within a set radius around the particle can be recorded over time and processed to yield the traces seen in Figure 3b–g. The applied voltage was kept low to avoid issues with solvent oxidation, fluorophore consumption, and degradation of the ITO surface. No fluorescence turn-on response is observed at particles in the absence of applied voltage or in cells that do not contain the LCO particles on the surface, though a decaying background is observed, likely due to bleaching of residual fluorescence from the nonlithiated HPNO dye. Additional evidence of lithium-ion release is provided by time-of-flight secondary ionization mass spectrometry (ToF-SIMS) maps that spatially resolve lithium-ion distribution before and after discharge. These controls, including examples of background fluorescence and photobleaching, are described in detail in the Supporting Information. The collected release traces of the LCO particles, including those in Figure 3, show a conspicuous diversity of responses, from no response at all, to short spurts of lithium release, to sustained fluorescence responses lasting many minutes. This diversity is significantly greater than what has been observed for the discharge dynamics of single LCO nanocrystallites.^37^

**Figure 3:**
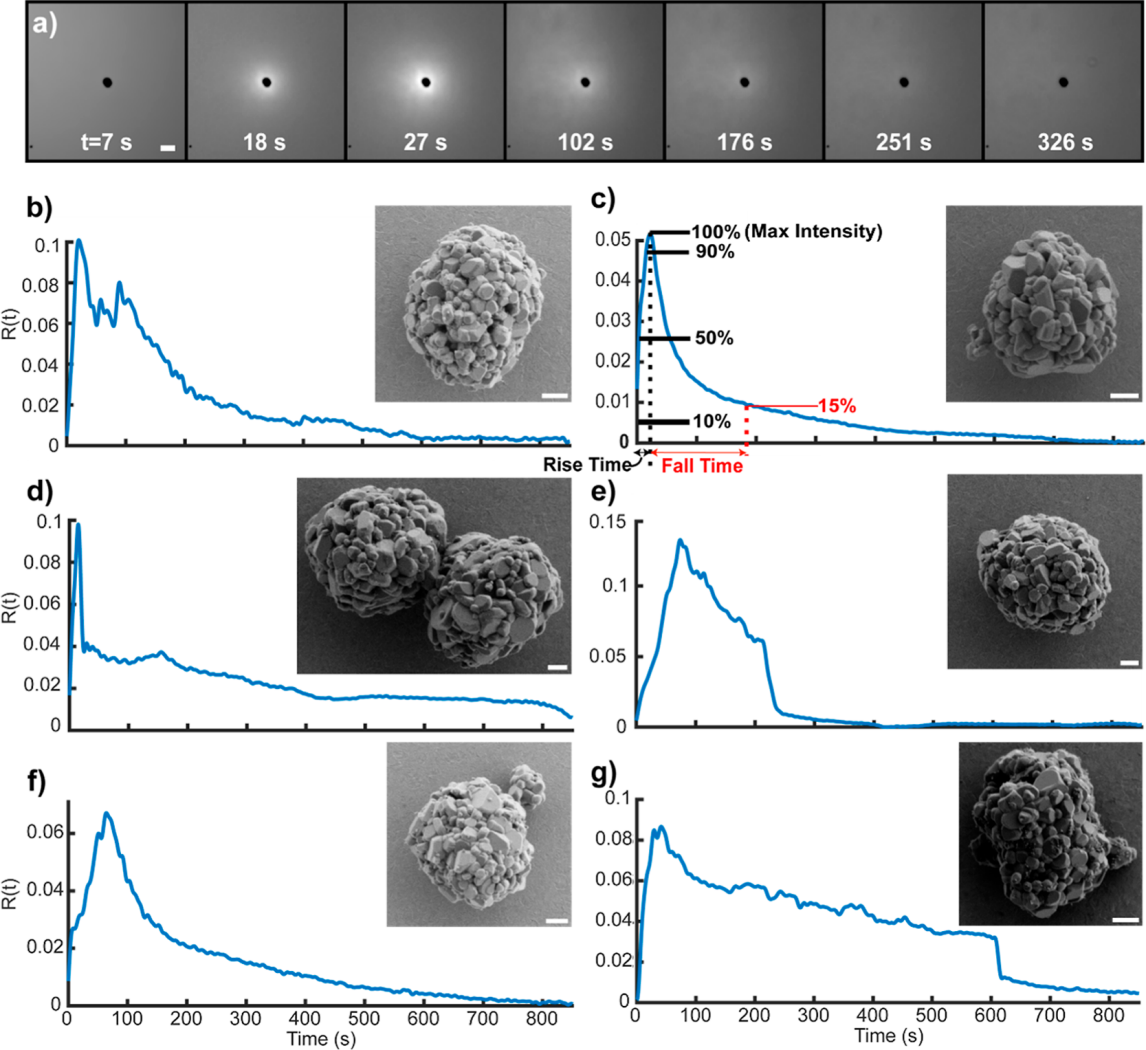
(a) Progression of a particle releasing lithium over 900 s. At *t* = 0, no voltage has been applied and no fluorescence above background is observed. By the first camera exposure, the applied potential has reached 1 V. By *t* = 18 s, the fluorescence “turns on” as lithium is released in response to the applied voltage. This voltage is held for the full 15 min of the experiment, but this small particle has exhausted its supply of lithium before the end, marked by a return to background intensity around the particle. Scalebar 10 μm. The corresponding release function is shown in panel (b). (b–g), Release functions (*R*(*t*), post-deconvolution) of individual LCO particles. (c) Example showing quantities extracted from fluorescence traces. Intensities reached at 10, 50, 90, and 100% of maximum intensity are recorded, along with the time taken to reach these values (shown in black text). Additional features independent of rise time such as the time to fall from the max down to 15% intensity can also be extracted (shown in red). Insets show the corresponding SEM images of each particle (1 μm scalebars for all insets).

### Processing of Fluorescence Traces to Reveal Single-Particle Dynamics

Fluorescence intensity traces for every particle are generated by averaging the intensity in a circular ROI (typically 12.5 μm) around the particle in each video frame. Black pixels corresponding to the particle area itself are excluded. Background subtraction is then applied by subtracting intensity measured from ROIs which are remote from the particle under study but under a similar illumination intensity. Each particle in the data set is subject to slightly different irradiance, owing to variation among the particles’ positions within the field of illumination. To account for this, observed intensities are also scaled to remove the influence of varied illumination among particles in the data set. More information on pre-deconvolution processing is included in the Supporting Information.

Observed fluorescence dynamics due to lithium mass transport at each ROI will include contributions from Li^+^ ions being released from the LCO particle into the volume, as well as solution-phase diffusion of Li^+^ ions out of the ROI and into the bulk solution. These processes happen simultaneously, with the observed experimental function, *E*(*t*), being a convolution (see Figure S13) of the lithium release function that describes discharge of lithium from the LCO cathode particle, *R*(*t*), and the diffusion function, *D*(*t*)­

E(t)=R(t)⊗D(t)

Determination of the release function, an intrinsic property of the individual LCO particle, is desired, while the diffusion function can be simulated (see Experimental Methods). The release function can be deconvolved from the observed experimental function using forward and inverse fast Fourier transforms (FFT), as given in the equation

$$R\left(t\right)={FFT}^{-1}\left\{\frac{FFT\left(E\left(t\right)\right)}{FFT\left(D\left(t\right)\right)}\right\}$$

This process is repeated for each fluorescence trace in the data set, yielding a large family of release function traces describing lithium-ion release dynamics from each of the individual LCO particles.

Comparison of each particle’s lithium release function is facilitated by choosing a succinct set of numerical parameters to describe each trace, as shown in Figure 3c. Some of these parameters directly yield kinetic information, such as the rise or fall times to fixed percentages of the peak amplitude. Other parameters include both kinetic and thermodynamic information, such as the peak amplitude, which is proportional to the local maximum lithium concentration. These parameters can then be used to summarize a given particle’s behavior and, as described below, explore potential correlations between release function and the morphology of the particle as discerned from SEM.

### Quantification of Delithiation and Lithium Ion Diffusion via Confocal Microscopy

To quantitatively convert fluorescence intensities into a direct measure of moles of released lithium ions, we deployed a Nikon Ti_2_ Yokogawa W1 spinning disk confocal microscope (405 nm ex, Plan Fluor 40× oil obj, NA = 1.30, Ziva CSU 445/49 BP filter, voxel size: 550 nm × 550 nm × 780 nm) which allowed for imaging at adequate speeds to monitor Li dynamics. LCO particles were exposed to 1.5 V vs the reference electrode over a 5 min period to track Li^+^ ion release. Confocal imaging ensured there was a submicron, consistent excitation volume across measurements, enabling observation of asymmetry of lithium ion release from different sides of a mesoparticle (see Supporting Information). Confocal imaging also opens a path to 3D imaging of release dynamics from throughout the mesoparticle, and even super-resolution microscopy with subdiffraction limited spatial resolution.^56^

Importantly, use of a known, constant excitation volume allows for fluorescence intensities to be directly correlated to their corresponding HPNO–Li concentration using a calibration curve (see Supporting Information). Li^+^ ion concentration and subsequent molar quantities could then be determined at individual voxels. To calculate the total amount of Li released, the molar release curve was integrated over the entire release time. Based on previous diffusion measurements^14^ we estimated that after acquisition of each frame (200 ms exposure), Li^+^ ions should quickly diffuse out of focus, eliminating the possibility of double-counting. The total charge passed at the particle can also be calculated, using the +1 charge of the Li^+^ ion. Plots of moles of Li^+^ released over time and the total moles of Li^+^ released are shown in Figure 4a,b.

**Figure 4:**
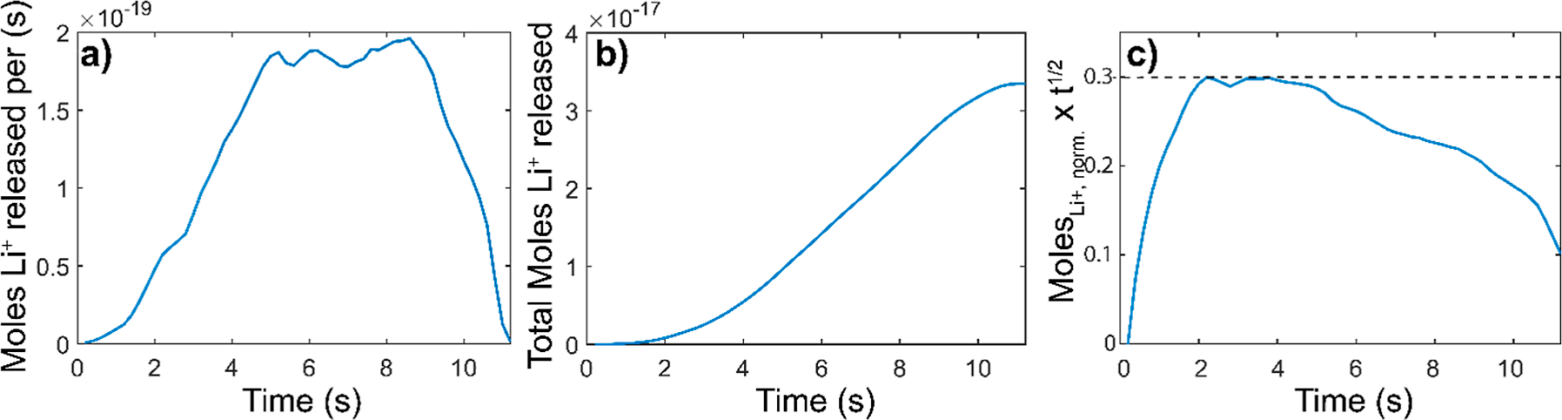
Analysis of Li^+^ transport from confocal imaging on single LCO particles. (a) Example release trace after calibration to show rate of Li^+^ release from a single mesoparticle. (b) Total amount of Li^+^ released as a function of time for one mesoparticle. (c) Plot modeled after ref 57 that demonstrates Cottrell conditions are reached from 2.2 s to 5.2 s (dashed line).

To measure the proportion of Li^+^ released from LCO particles, we estimated the size of each particle and used the tap density provided on its product data sheet (2.6 g/mL) to estimate the total Li content within the particle. Across the particles analyzed confocally we found that the particles released between 0.5% and 1.1% of their total Li content upon application of a 1.5 V potential. This observed change in lithium content corresponds to directly observing the initial 1–2% of the discharging process, assuming that a particle is still 40% lithiated^57^ when a lithium-ion battery using an LCO cathode is at 100% state of charge (SOC). While this is a small portion of the delithiation that occurs over a full charging cycle, the kinetics of this initial release are still significant for understanding the performance of these materials.

Our direct measurement of Li^+^ concentration with respect to time allowed us to further model the depletion kinetics of the LCO particle. Following previous analysis,^58,59^ we applied the Cottrell equation with planar boundary conditions. A plot of fraction of moles Li^+^ multiplied by *t* ^1/2^ vs *t*, an optically derived analog to plotting *I*_(*t*)_ *t* ^1/2^ vs t, is shown in Figure 4c. The plot reveals the different diffusional time scales that contribute to the observed release traces, with a Cottrell region obtained as a plateau at short times (2–4 s). Planar boundary conditions were selected because across the limited voxel depth of 780 nm, the LCO particle will appear planar. In this region, a Li^+^ diffusion coefficient of 1.5 × 10^–8^ cm^2^/s was obtained, which is comparable to previously obtained values for LCO mesoparticles.^60^

### SEM Imaging of LCO Particles

As shown in Figure 3, a wide diversity of release functions was observed. The origin of this diversity is of substantial mechanistic interest. To investigate whether morphological differences of these particles were the origin of the release function diversity, SEM micrographs were acquired for all LCO particles after depositing them onto the ITO/glass surfaces, but before electrochemical investigations, to allow correlative fluorescence/SEM imaging on each particle. SEM micrographs of the particles depicted in Figure 3b–g are shown as insets.

These SEM images reveal that the LCO particles are all aggregates of smaller particles. Going forward, we will refer to these aggregates as “mesoparticles,” while referring to the component smaller particles as “nanocrystallites”; it is these nanocrystallites which have been the subject of several previous single-particle studies.^22,32–34,37^ While the mesoparticles are not single crystals of LCO, they are reflective of the varied arrangements of LCO within an operating lithium-ion battery, as summarized in Figure 1. Commercial LCO electrodes are indeed composed of densely packed clusters of many LCO nanocrystallites, unlike the larger individual crystals often studied in the literature. A tremendous diversity of particle geometries is present across our samples, and even similarly sized mesoparticles appear to differ in the number, orientation, and size of their constituent nanocrystallites. As both the particle morphology, as discerned via SEM, and the measured lithium-ion release functions, as obtained via our new fluorescence method, are both highly varied in nature, we sought to examine if the differences in observed lithium release dynamics are correlated to any quantifiable physical aspects of the mesoparticles as determined from SEM.

To this end, the SEM data were processed using a custom image analysis protocol written in ImageJ and MATLAB^61–63^ that extracted regions describing the size and shape of each of the mesoparticles along with their component individual nanocrystallites. This analysis is described in greater detail in the Supporting Information, including Figure S16. Though this analysis only yields an approximation of each mesoparticle composition, since the SEM image is a 2D representation of a 3D particle, the composition of the particle faces being captured and analyzed provides a representative sample of the overall particle composition. The parameters extracted from SEM included global parameters such as the diameter, ellipticity, and visible surface area of the mesoparticle, as well as more microscopic features such as the number and dimensions of the component nanocrystallites. Again, a wide variety of values is observed, with mesoparticle sizes ranging from <1 μm to ∼15 μm in diameter, and anywhere from tens to hundreds of nanocrystallites visible within each cluster. The nanocrystallites themselves were seen to range from ∼300 nm to almost 1 μm in diameter, and while there was a slight correlation between nanocrystallite size and full mesoparticle size (See Supporting Information), examples of larger particles composed of many small nanocrystallites could be found, as could particles composed of only a few larger crystals. Summary statistics describing all of this variation were collected to form a data set of particles (*n* = 319), linking the morphology information on each mesoparticle from the SEM image analysis with the corresponding lithium release function extracted from its fluorescence data. Future implementations could also deploy more advanced mesoscale structural analysis, such as 3D electron tomography or element-specific imaging.^25,64–66^

### Relationships Between Mesoparticle Behavior and Structure

The data set was then used to create plots relating each morphology parameter with each lithium release parameter. The morphology parameters are the SEM-derived variables from the output of the image analysis protocol above. The lithium release parameters are those derived from the release traces deconvolved from the fluorescence measurement, illustrated in Figure 3. Because these correlations are hard to interpret visually, a simple linear regression was calculated for each set and used to derive a correlation coefficient and *p*-value for each set of variables. The result of this analysis and visualization is shown in Figure 5, with corresponding tables of correlation coefficients and *p*-values provided in the Supporting Information. Linear regressions were chosen, instead of nonlinear models which are not justified without a more detailed microscopic model. The range of *r* values obtained for the correlation plots displayed in Figure 5 is −0.15 to 0.63.

**Figure 5:**
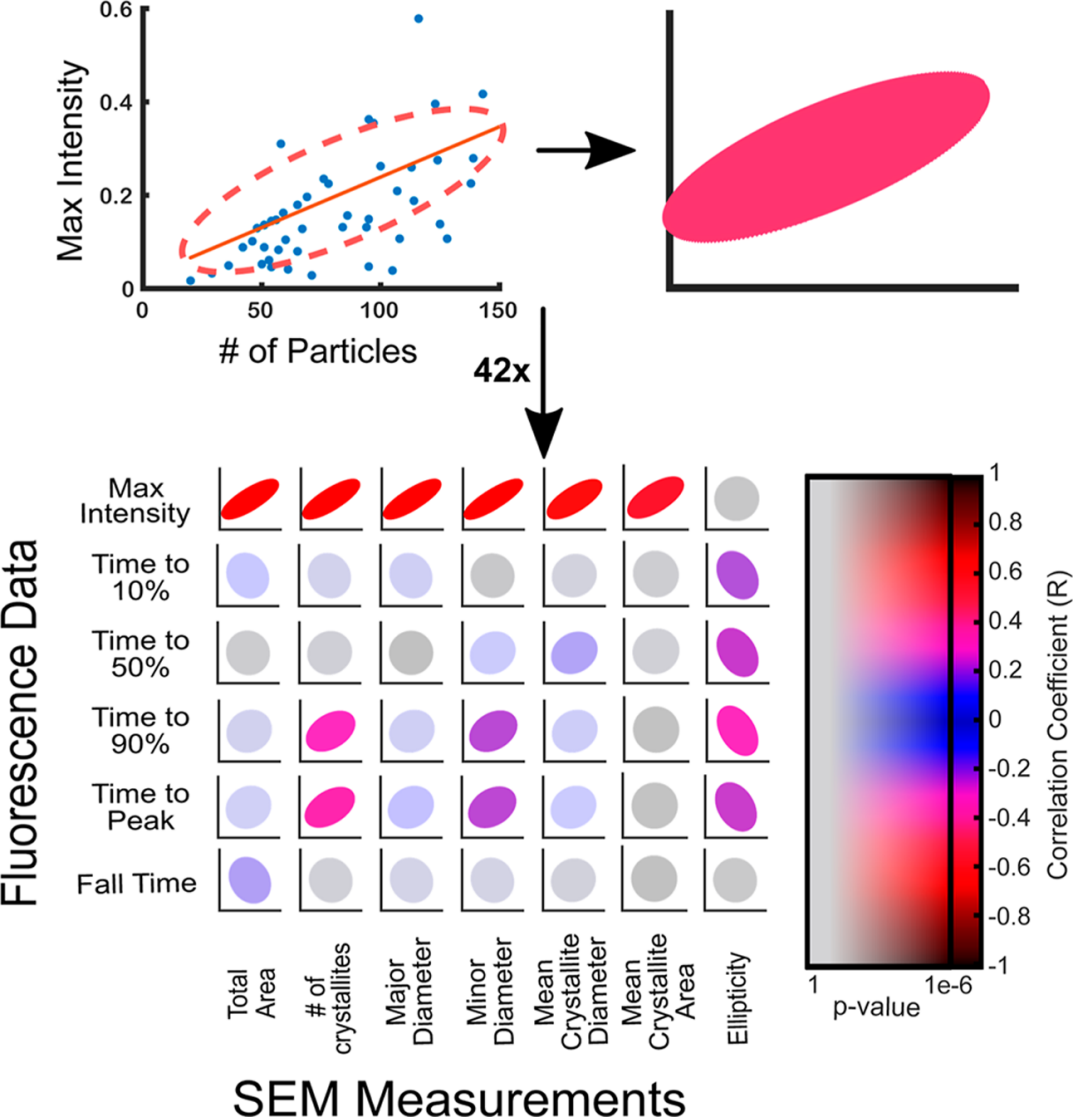
Visualization of correlation strength across variables. Scatter plots of variables derived from fluorescence information (left axis) and SEM information (bottom axis) were generated and fit linearly. Ellipses represent deviation from linearity, where a perfect circle represents no correlation, an ellipse is a stronger correlation, and a straight line is a perfect correlation (*R* = 1). Colors (cold to hot) represent this same distribution of correlations. Graphs of relationships that were deemed statistically insignificant are greyed out. Correlation coefficients (*R*) and *p*-values for all elements of the above matrix are represented by the 2D colorbar and numerical values are provided in the Supporting Information.

As stated before, many more parameters describing the SEM-derived measurements and lithium release behavior were extracted, but only parameters showing correlation and several select counterexamples are shown in Figure 5 (see Supporting Information for full analysis). The most striking aspect is the strong correlation of the maximum peak amplitude of *R*(*t*) (and therefore the maximum concentration of lithium around the particle) with almost every simple morphological parameter derived from the SEM images. It is worth noting that the SEM parameters describing area, number of nanocrystallites in the mesoparticle, and diameter are all correlated with one another (see Supporting Information), and so the displayed set of correlations is likely a manifestation of one dominant parameter such as the total particle size or number of nanocrystallites being correlated with peak amplitude. Integrated peak areas also show a similar correlation to peak maximum as expected, but the relationship is somewhat weaker, likely due to the residual contribution of background (see Supporting Information). Regardless, it is intuitive and encouraging to see larger particles exhibiting larger peaks, as the peak height is related to capacity, and so this correlation reflects the expected increase in lithium discharge capacity for larger particles.

Comparatively, the correlations related to kinetic properties (ie, the time it takes to reach a particular lithium release level) are weaker and exhibit some unexpected trends. The time to 10% and 50% release exhibit nonexistent or weak correlations with various structural parameters. However, somewhat stronger correlations emerge at time to 90% and 100% of peak amplitude. Here, mesoparticles composed of larger numbers of nanocrystallites take longer to reach higher amplitudes of release function. When the minor diameter of the mesoparticle is large or the mesoparticle ellipticity is low, two situations that reflect more spherical mesoparticles, increased time to higher amplitudes of release function is also observed.

On the other hand, fall time exhibits no correlation with any of the examined structural parameters. We note that the contribution of solution-phase lithium ion diffusion has already been deconvolved from the empirical trace to produce the release functions in Figure 3, and so this transport process is expected to make a negligible contribution. The lack of any structural correlations suggests a different underlying mechanism for the discharge of later-arriving lithium ions.

Simultaneously examining the strong correlations of maximum intensity with size and the more moderate correlations of rate with mesoparticle shape enable more mechanistic insights about the discharge process. The observed peak amplitude is influenced by thermodynamic parameters like total capacity, but also kinetic parameters (the peak height will be larger if all of the lithium is released quickly due to the finite rate of diffusion). The weaker correlation between mesoparticle structure and the purely kinetic parameters (such as time to a percentage of the peak maximum) suggests that the correlation with peak height is dominated by the capacity.

Taken together, a refined mechanistic picture can be assembled. The rate of release of the first lithium ions by the mesoparticle is relatively independent of mesoparticle structure: this observation makes sense since these early release ions are likely coming from nanocrystallites close to the ITO contact and the mesoparticle surface. However, to get to the maximum release function amplitude, nanocrystallites from throughout the mesoparticle must be oxidized, a process that is expected to be slower when the total number of nanocrystallites is larger or the overall mesoparticle is more spherical, thus having lower surface to volume ratio. For these larger mesoparticles, oxidation and release rely on an increasingly long and circuitous chain of electrochemical contacts to connect all of the constituent particles, as further discussed below.

Overall, the weaker correlations of structural parameters with kinetic behavior and conspicuous diversity in release function shapes is interesting and unexpected, particularly when single-nanocrystallite experiments focus on the kinetics of intraparticle diffusion of lithium, which would suggest a strong correlation with surface area-to-volume ratio. One possibility is variability in the resistance of the surface conductor. It is known that thin layers of ITO can exhibit differences in conductivity, including sample-to-sample variability from the same lot, and even spatial differences in the same substrate.^67–69^ Analogously in silicon nanowire particles, differences in the resistance of the underlying nanowire framework were hypothesized to be responsible for differences in velocity of reaction fronts in the individual particle delithiation.^19^ Thus, surface variability of conductivity is likely a contributor, with varied responses even from mesoparticles located very close to one another on the ITO surface being observed. Though shelf-stability varies among ITO coatings from different sources, eventual aging of the ITO coating can also lead to changes in conductivity across the surface. To mitigate the influence of aging, newly coated ITO coverslips were ordered regularly and used soon after ordering, and unused coverslips were stored in sealed air-free containers at low temperature. Major aging effects were not observed in the ITO substrates used in the present study.

A second possibility is that even if voltage is applied evenly across the ITO surface, the differing morphology of the mesoparticles themselves may cause differing contact resistance with the ITO surface, and therefore differing influence of the applied voltage. These diversities of contact and electrode resistances would also be present in the full manufactured device, and so may be reflective of behavior seen in real-world battery applications. Some analysis of the influence of contact resistance between the electrode particle and the current collector on power output demonstrate that the contact resistance may account for as much as 25% of cell polarization, though it should be noted that this is an upper limit and was determined specifically for high current applications for a set of lithium ion battery materials that does not include LCO.^70^ In our data 53% of mesoparticles observed via SEM did not show a fluorescent response, suggesting that this fraction likely did not possess good contact with the underlying ITO. However, the strong correlation between mesoparticle size and peak intensity suggests that as long as a minimum contact fidelity is achieved to trigger dynamics, variations in contact or electrode resistance in this system are not of sufficient magnitude to reduce the voltage drop across the mesoparticle and decrease observed capacity. Otherwise, the correlations between size and peak intensity would be substantially weakened. Further, the nonmonotonic and nonself-similar nature of the release functions (Figure 3) suggests greater underlying diversity than could be caused solely by differences in contact resistance to the ITO.

The remaining source of intrinsic variability in release traces likely derives from the internal physiochemical contacts between the individual nanocrystallites within the mesoparticle, as mentioned above in the context of correlation with kinetic behavior. In fact, Wheeler et al. demonstrated a need for their models of LCO electrodes to incorporate a distribution of particle–particle contact resistances in order to properly capture the performance of bulk measurements of electrodes.^42^ Other models of lithium-ion electrodes also needed a distribution of these interparticle contact resistances between particles in order to correctly model the system behavior.^43^ Additional experiments by Morelly et al. indirectly revealed that short-range electronic contacts play a decisive role in the performance of composite battery electrodes by tuning the amount of conductive carbon additive present.^44^ The results presented in this work constitute the first direct experimental observation of the mesoscale impact of underlying physiochemical diversity.

These simulations^42,43^ and bulk experiments^44^ suggest that the connections between nanocrystallites, so significant for modeling the observed cell performance, are likely a major contributing factor in shaping the release traces that we observe experimentally. The varied internal connections between nanocrystallites within the mesoparticle give rise to complex patterns of release, leading to lithium release functions with general behavior only somewhat influenced by—and details mostly independent of—the shape and size parameters of the mesoparticle, as observed in our work. In this context, the variability in observed fluorescence traces echoes the variety of parameters required by the bulk models. While these simulations conclusively show the existence of this diversity in internal resistance, the problem is underdetermined at the bulk level: simple model distributions are used in these studies to fit the data since the true distribution cannot be uniquely estimated from the bulk data. However, mesoscale systems such as those shown here, where the morphology of the mesoparticle and component nanocrystallites are known, represent a system that contains a key element of complexity not found in single crystallite systems, but is also tractable. Refinement of models, such as in Ref 42 that can capture the diversity observed in these mesoscale systems by adding time-resolved lithium ion release data from tractable mesoscale systems is a potentially impactful application of the method developed here.

## Conclusions

In summary, we report a method for imaging the release of lithium ions from individual battery cathode mesoparticles using fluorescence microscopy, with spatial and temporal resolution. Application of our method allowed the direct observation of the substantial variability in mesoparticle kinetic behavior. Correlative SEM imaging and analysis allowed correlation of observed behavior with morphology of the nanocrystallites composing the mesoparticle. This technique is shown to be useful as an in situ technique, capturing the diversity of behavior found in more complex particles at the mesoscale regime between the single-crystal and whole-electrode limits. This approach is not specific to the cathode material chosen and does not require phase changes resulting in optical or diffractive contrast differences, and so should have general applicability to the study of many lithium-ion battery materials.

Comparison with SEM micrographs shows the existence of correlations between mesoparticle size and lithium ion capacity, as expected, but conspicuously weaker correlations between morphology and discharge kinetics. Prior models suggest that diversity in nanocrystallite–nanocrystallite contacts accounts for some observed cell performance seen at the bulk level, but our work for the first time explicitly captures the dynamics caused by this diversity by enabling observations at the mesoscopic level. Indeed, this work describes a substantial and previously inaccessible source of data on mesoscale behavior of battery materials, a key length scale affecting performance, and allows for further refinement of system-wide models.

## Supplementary Material





## Notes

Detailed experimental and analysis procedures, HPNO synthesis, SEM characterization of mesoparticles, optical setup, electrochemical cell, ROI selection and background subtraction, deconvolution, comparison of traces, SEM image analysis, histograms of particle behaviors, statistical analysis of correlations, expanded data on correlations, diffusion constant determination, control experiments, cyclic voltammogram, ToF-SIMS mapping, lithium concentration calibration, galvanostatic experiments (PDF)
Supporting Information movie: a video showing fluorescence imaging of release of lithium from the particle featured in Figure 3a (AVI)
