# Supplementary material for: Imaging of Lithium Ion Release from Individual Cathode Particles Shows Evidence for Diversity of Intraparticle Contacts
Source: Chem Mater. 2025 Jul 17;37(15):5785–95. doi: 10.1021/acs.chemmater.5c00906 (PMC12506632; doi:10.1021/acs.chemmater.5c00906)
Supplement: Supplementary file 1 [file cm5c00906_si_001.pdf]

Methods and Supplementary Information for

Imaging of Lithium Ion Release from Individual Cathode Particles Shows Evidence for Diversity of Intraparticle Contacts

Andrew C. Cavell[†], Evan T. Jensen[†], Benjamin A. Brewster[†], Mengcheng Wu[†], Mackinsey A. Smith[†], Michael S. Mattei[†], Rachel Czerwinski[†], Lucy C. Kneeley[†], Michael S. Foy[†], Aisley F. Fleming[†], Elisa T. Harrison[§], Sabrina L. Peczonczyk[§], Alvaro G. Masias[§], Randall H. Goldsmith[†]

[†]Department of Chemistry, University of Wisconsin-Madison, 1101 University Ave., Madison, Wisconsin 53705, United States

[§]Ford Motor Company, 2101 Village Road, Dearborn, Michigan 48121, United States

LCO Sample Preparation and Deposition.

LCO powder was used as received (electrodesandmore.com, BLCOP-25). The conductive transparent coverslips used were Indium Tin Oxide (ITO) coated glass coverslips sourced from Delta Technologies (CB-50IN-0105, 5–15-ohm resistance, 120-160 nm ITO thickness, 0.15 mm glass thickness). The LCO powder was suspended in isopropyl alcohol (2-propanol HPLC Grade, Fisher), and this suspension was used for sample deposition. The suspensions were created by serial dilution to 1 mg LCO/ 1 mL of 2-propanol and 0.25 mg LCO/ 1 mL of 2-propanol. All glassware used in this process was first rinsed thoroughly with HPLC solvent, dried, and then cleaned in a plasma etcher for 10 minutes at 300W to remove potential impurities from the glassware. ITO coverslips were etched by hand using a diamond scribe in an asymmetric pattern to help identify particles in imaging (see SEM imaging section below). The suspensions were agitated by hand to promote uniform concentration immediately before depositing 80 μL of LCO/IPA on an ITO coverslip and spin coating at 4000 rpm for 30 seconds.

Synthesis of 2-(2-hydroxyphenyl)-naphthoxazole (HPNO)

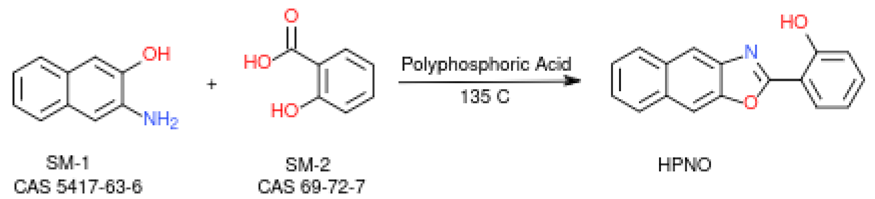

Figure S1. Scheme of HPNO synthesis.

2-(2-hydroxyphenyl)-naphthoxazole (HPNO) was synthesized according to a modified literature procedure.[1] Salicylic acid (2-hydroxybenzoic acid, 1.00 g, 6.28 mmol), 3-amino-2-naphthol (887 mg, 6.28 mmol), and 50 g of polyphosphoric acid were added to a 100 mL round bottom flask. The reaction flask was then heated in an oil bath to 135 °C and stirred under nitrogen atmosphere for 4.5 h. After 4.5 hr the

flask was removed from heat and allowed to cool partially before quenching with water. The solid product was collected and washed with saturated NH_4Cl and water, dried overnight, and then purified by silica column chromatography using 20:80 ethyl acetate:hexanes. The product was further purified by trituration with pentane before use in optical experiments, after which 200 mg of product (12% yield) were recovered. Spectroscopic details are available in the prior literature. ^1H NMR (400 MHz, $CDCl_3$): δ 8.17 (1H, s), 8.10 (1H, dd, J = 1.7 Hz, J = 7.8 Hz), 8.03−7.97 (3H, overlapping), 7.54−7.47 (3H, overlapping), 7.16 (1H, d, J = 8.6 Hz,), 7.05 (1H, t, J = 7.5 Hz). ^{13}C NMR (100 MHz, $CDCl_3$): δ 164.9, 159.4, 148.0, 139.8, 134.2, 131.7, 131.6, 128.5, 128.0, 127.6, 125.7, 125.0, 119.7, 117.5, 116.5, 110.3, 110.0, 106.5. MS-ESI+ (m/z): $[M + H]^+$ calcd for $C_{23}H_{15}N_2O_3$: 237.27; found, 237.26.

HPNO Solution Preparation.

All chemical reagents were purchased from Sigma - Aldrich unless otherwise specified. A 25:1 v/v solution of propylene carbonate (PC) (Sigma - Aldrich 310328 - 500mL) and triethylamine (TEA) (Sigma - Aldrich T0886 – 500 mL) was first prepared. 0.20 g of tetrabutylammonium hexafluorophosphate ($TBAPF_6$) was weighed and added to the solution to create a 0.1 M electrolyte solution. Finally, a 3.8 mM solution of HPNO was created by adding 4 mg of HPNO powder to 4 mL of the $TBAPF_6$/TEA/PC solution. The solution was sonicated for 90 minutes and allowed to sit overnight to dissolve fully. It was then filtered through a Whatman 0.2 µm TF filter to remove undissolved HPNO or other precipitates that could interfere with imaging. The solution was stored at room temperature until use.

Scanning Electron Micrograph (SEM) measurements

A Zeiss Supra 55-VP FEGSEM was used to image the ITO coverslips with deposited LCO particles. 4 samples are attached to a PELCO SEMClip, 63 mm 4 clip pin mount stage and the particles were imaged with an EHT gun strength of 2.0 kW and a working distance of around 7.1 mm. Images were taken using a line averaging technique. Images of individual particles were taken in addition to images of a roughly 50 um x 50 um field of view of the particles of interest.

Samples are randomly distributed due to the spin coating process. In order to locate the same particles repeatedly, a specific pattern of scores was made using a diamond scribe in order to aid navigation on both the SEM and fluorescence microscope. Particles of interest were always taken from the area underneath the second score from the left in the row of 4 scores. SEM imaging was done prior to electrochemistry in order to get a clear image of particle morphology before contact with electrolyte solutions. SEM images taken after electrochemical experiments are dominated by leftover deposits from the dried electrolyte solution, and as a consequence, discerning detail of the LCO particles is difficult (Figure S5).

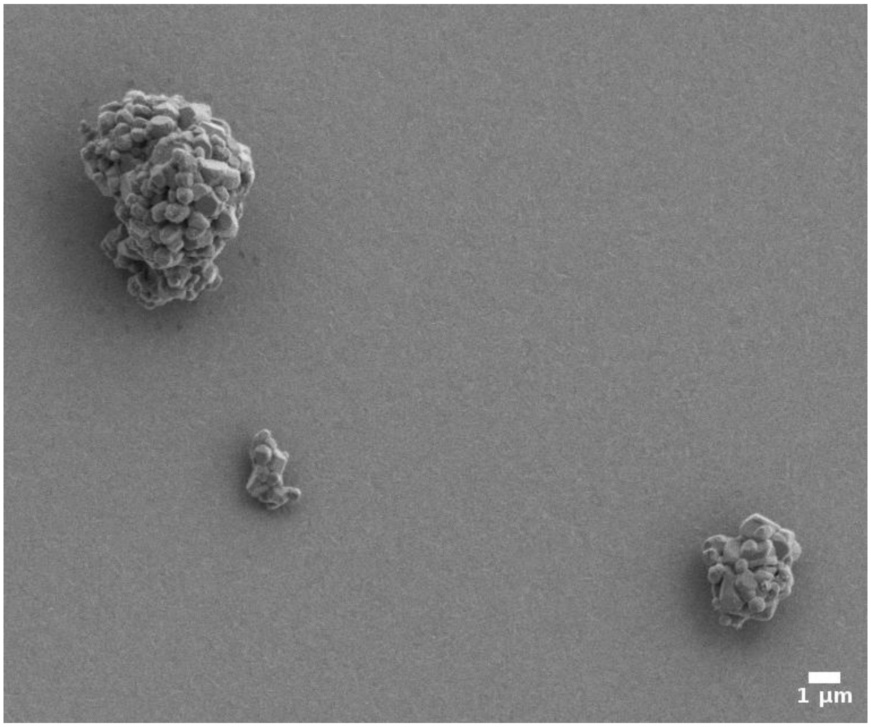

Figure S2. SEM micrograph of LCO particles on an ITO/glass surface.

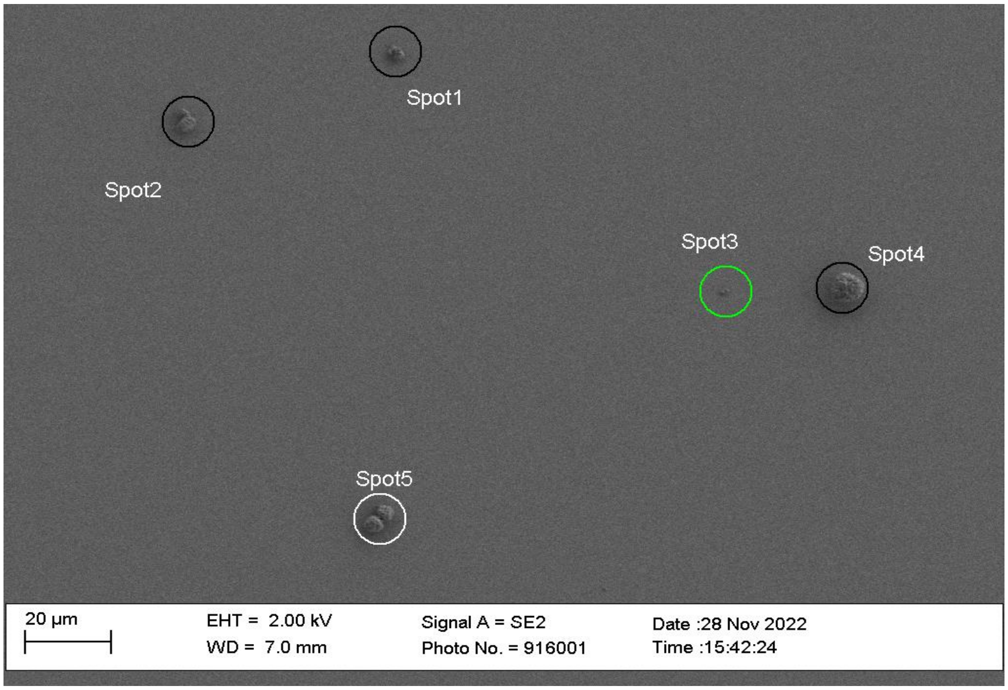

Figure S3. SEM image of a region with 5 LCO particles identified on an ITO coverslip.

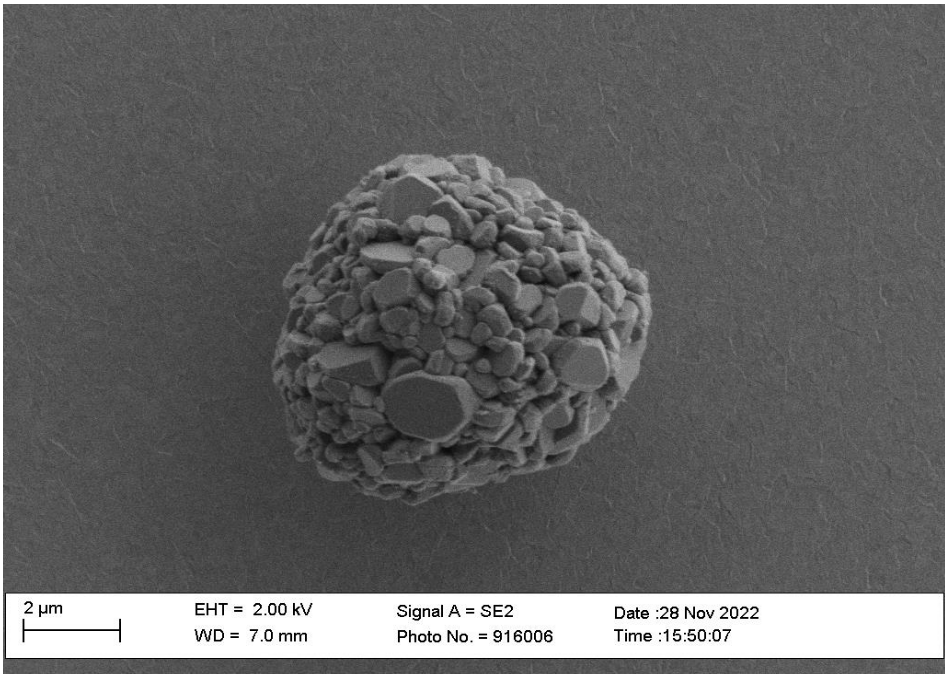

Figure S4. Zoomed in SEM of an LCO particle isolated from the previous image.

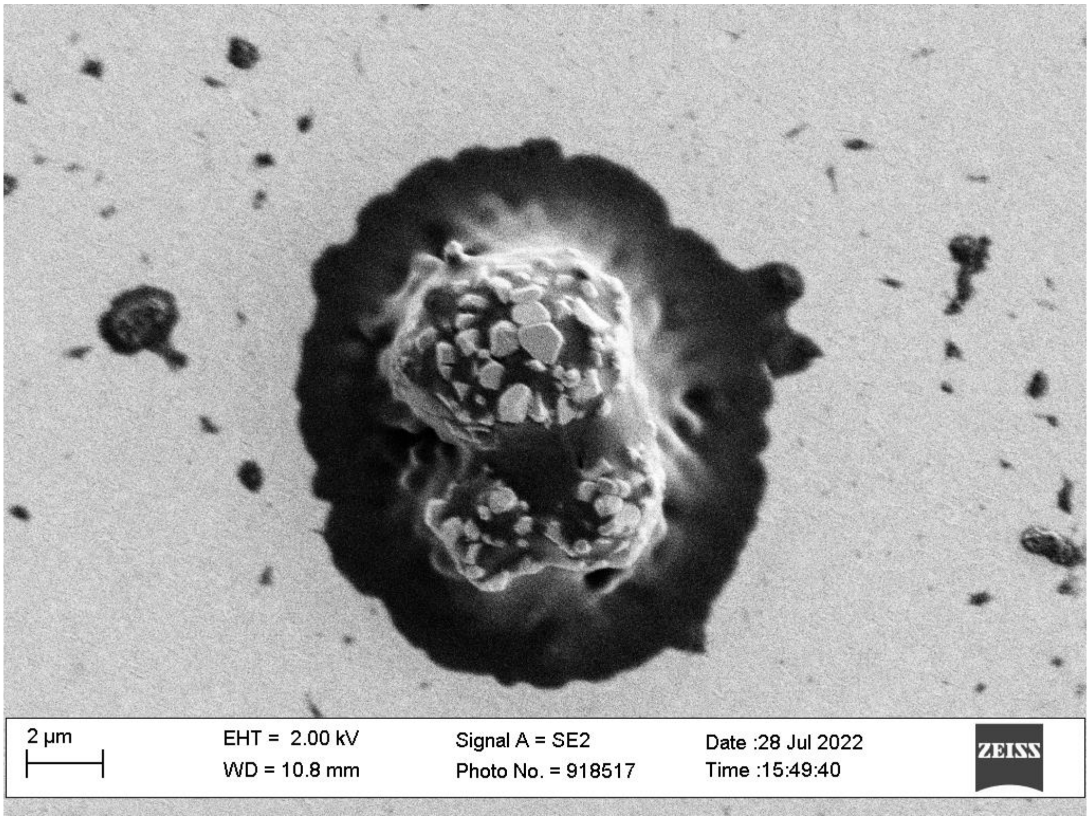

Figure S5. SEM images taken after experiments show residue that prevents clean image analysis.

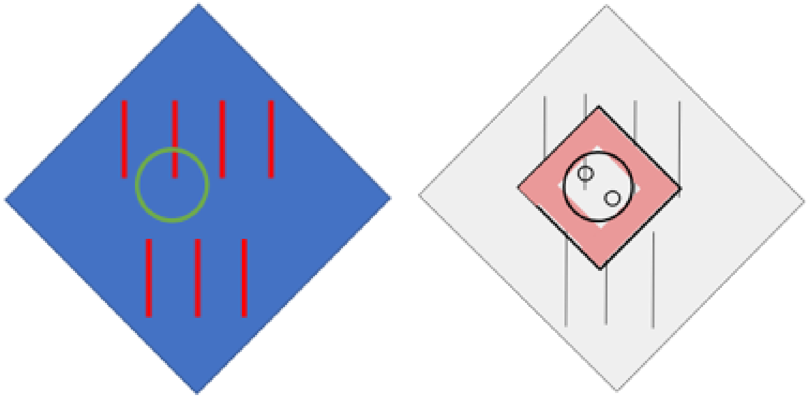

Figure S6. Scoring pattern used to locate regions of a sample reproducibly. Left: Red lines are score marks; the green circle describes the region examined by SEM to find particles. Right: This area is later covered by a chamber for electrochemical experiments and the same particles located via optical microscopy.

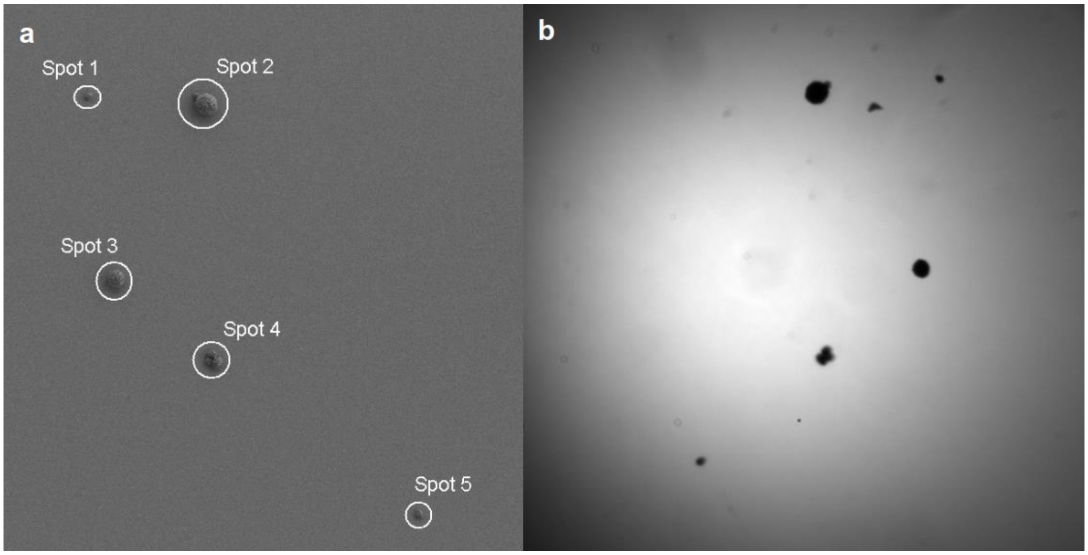

Figure S7. The same group of particles is visible using a) SEM and b) fluorescence microscopy. The apparent flip between the images is due to the use of an inverted microscope (imaging through the glass bottom of the sample) for the fluorescence image, while the SEM image is taken from above.

Optical Setup for Fluorescence Microscopy

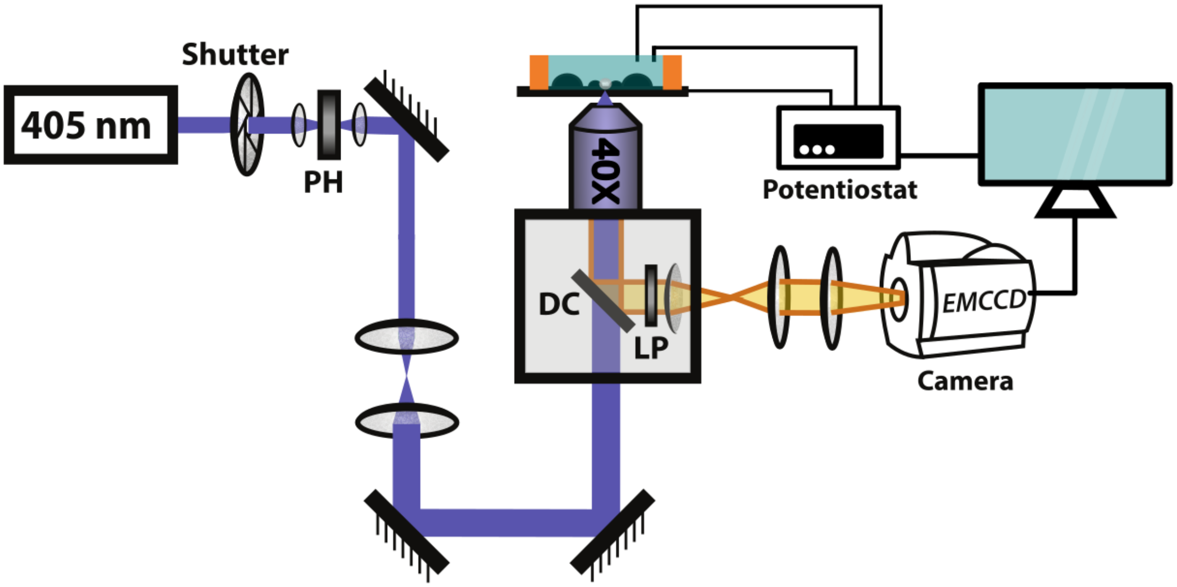

Figure S8. Optical setup for fluorescence measurements. DC: dichroic. PH: Pinhole. LP: Longpass filter

Microscopy was performed on an inverted microscope (Nikon TE-2000U), with illumination provided by a 405 nm laser (Cobolt MLD, 120 mW) for excitation of the HPNO dye. Due to the uneven beam profile typical of these diodes, spatial mode cleanup was achieved by focusing through a 50 μm pinhole to provide a symmetric and round beam. The laser was attenuated with ND filters (OD1.8 in total), then aligned with a mirror relay through a telescope to expand the beam to fill the widefield lens. The widefield lens focuses to the back of the objective (Nikon 40x, 0.65 NA). An electronic shutter (Uniblitz) at the beginning of the beam path was used to keep the sample from being exposed to excitation light prior to imaging, in order to prevent photobleaching. Emission was separated from the excitation light using a 405nm long pass dichroic beamsplitter (Semrock Di02-R405-25x36). Emission from the sample was collected by that same objective, passed through a 408 nm Long Pass wavelength filter (Semrock BLP01-405R-25), and directed to the collection optics. Emission was collected and recorded on an EM-CCD camera (Andor, Ixon 897). Custom labview code interfaced with the camera and synchronized the shutter, camera acquisition, and potentiostat. Unless stated otherwise, each experiment used a 100 ms exposure time. The CCD was cooled to -80C during data acquisition. Frames were taken consecutively for up to 6900 repetitions to acquire up to 15-minute-long videos for each experiment at a frame rate of 7.56 frames per second.

Electrochemical Experimental Details

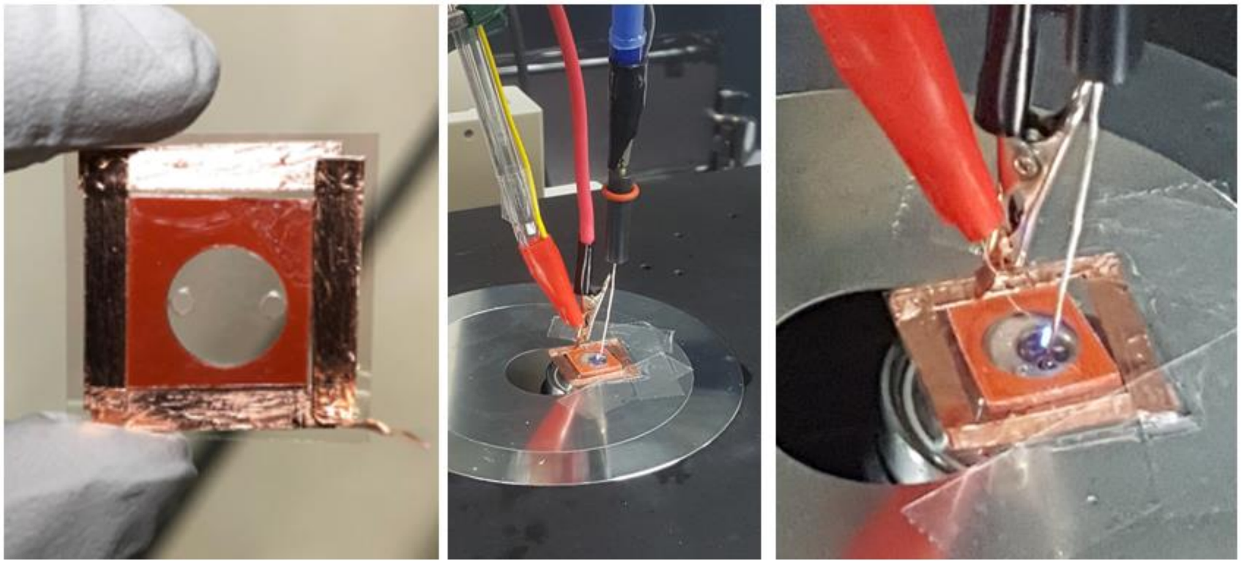

Figure S9. Images of the cell used in electrochemical experiments. Left, the assembled cell with chamber adhered and copper tape applied to afford contact with the ITO surface. Center, the cell atop the microscope stage with counter and reference electrodes in place. Right, closeup image of the cell with electrodes in place and connection made to the ITO surface – light blue fluorescence can be seen at the center of the sample above the center of the objective lens.

All electrochemical experiments were performed using a CH Instruments 620E potentiostat. All experiments were conducted as controlled-potential coulometry experiments with a 3-electrode cell in which the ITO coverslip acted as a working electrode, a silver wire was used as a reference electrode, and a platinum wire was used as the counter electrode. Prior to each acquisition, the electrodes were rinsed with isopropyl alcohol and polished with diamond lapping sheets (Thorlabs Polishing Sheets) to ensure a clean surface, then rinsed again.

The electrochemical cells were constructed by adhering individual SecureSeal™ Hybridization Chambers (Grace Bio-Labs) to the ITO coverslips and surrounding the outside with copper tape to ensure good contact with the ITO. After placement on the microscope stage, the chambers were filled with the HPNO solution described above, and the counter and reference electrodes were inserted into the solution through each of the ports on the top of the chamber. Contact with the ITO was made by connection to a tag of copper tape coming off of the surface (see Figure S9).

Custom LabVIEW code was written to synchronize the camera acquisition with the electrochemical experiment in order to reproducibly begin the experiments at the same point in the video. This code also served to independently record the voltage and current at the cell.

Fluorescence Trace Analysis

For each fluorescence experiment, custom MATLAB software is used to extract a trace of fluorescence vs time for each particle. This is an automated process, requiring only that the user select the positions of the particles in the video. The code automatically extracts the fluorescence over time in a region of interest (ROI) around the particle, and additionally finds an ROI with comparable illumination intensity to use for background subtraction (i.e. one that has similar intensity in the video at t=0). In this work we consistently use an ROI diameter of 12.5 μm, as this is large enough to encompass most of the particles, but still small enough to achieve a good signal to background ratio.

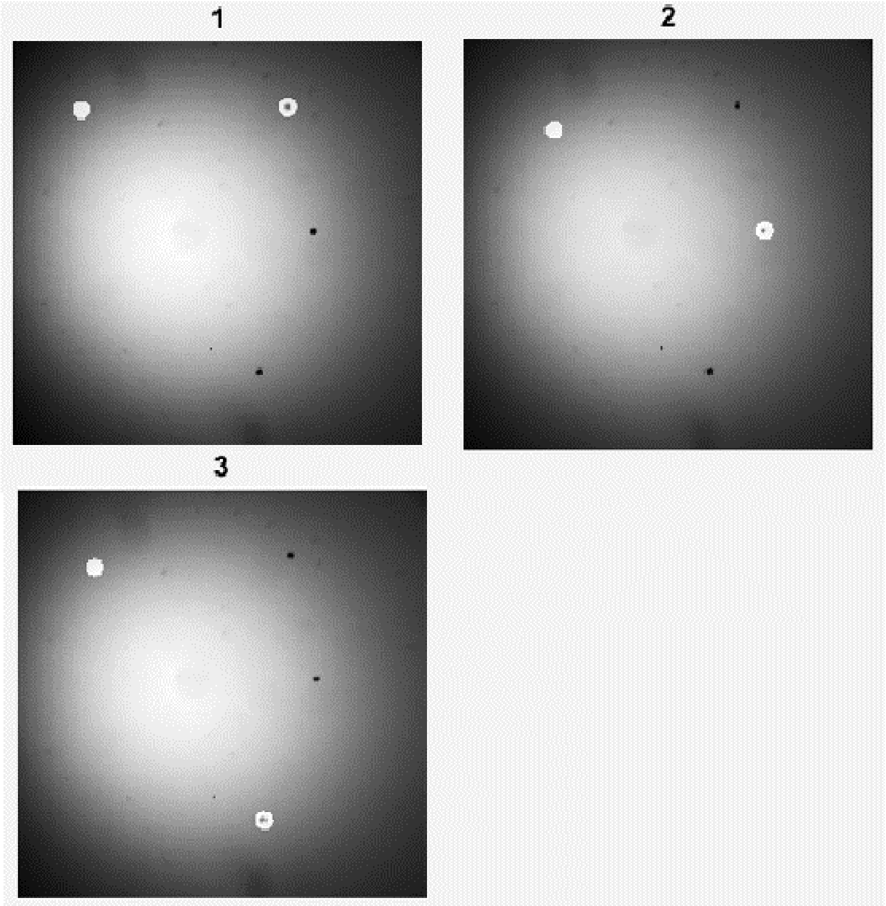

Figure S10. Example output frame from the MATLAB analysis. 1, 2, and 3 all show the same video frame, with different areas highlighted. The user has selected the locations of 3 particles (each particle shown highlighted in images 1, 2, and 3), and the software has found ROIs to be used for proper background subtraction (empty ROIs highlighted in each image).

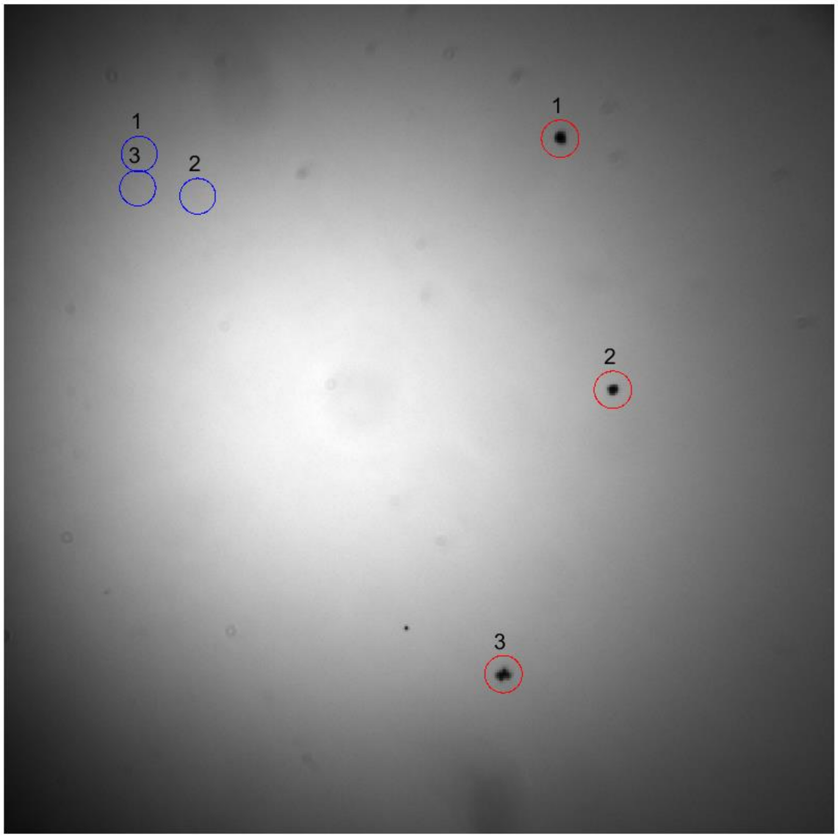

Figure S11. Diagram of ROIs chosen by user (Particles 1-3, red circles), and those automatically assigned for measurement of background by the MATLAB code (empty blue circles 1-3). The code ensures that the background ROIs chosen are similar in illumination intensity to the particle ROIs, while being remote as possible from the particles of interest to prevent bleeding of signal into the background measurement.

Observed background from HPNO profluorophore in solution is quite high at the start of each experiment and requires careful background subtraction, thus motivating the need for an automated procedure that is consistent in its handling of the data. As seen in Figure S12, while the background is typically high at the beginning of the experiment, it is observed to lessen throughout the experiment. Further examination of this behavior in control experiments is given later in the SI. In addition to the background subtraction, the data is also normalized (by division of the trace by its initial value) to reduce the influence of variability in sample geometry or preparation. This process ensures that all traces can be compared to each other, as the effects of background, uneven illumination, inconsistent profluorophore concentration, and photobleaching have all been mitigated, leaving only the influence of changing lithium concentration on the fluorescence signal. The code returns traces for each particle in the video, along with some summary information about the parameters used. The fluorescence traces are then used to obtain release traces via the deconvolution described in the main text and shown in Fig. S12d and S13 below. These are added to a database and used in further analysis.

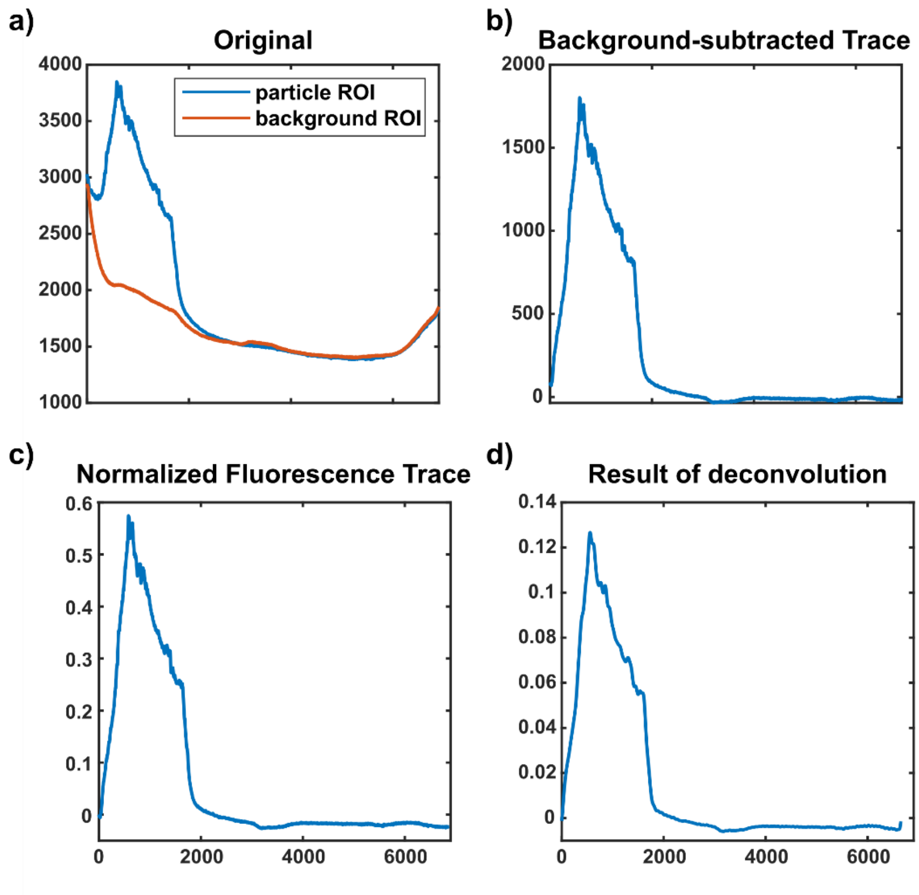

Figure S12. Processing of fluorescence data. Panel a) shows both the raw fluorescence intensity (integrated over the ROI) for the particle and the measured background. Background is significant at early times but drops quickly. Panel b) shows the result of background subtraction (the difference of the two curves shown in a). Panel c) shows the same background-subtracted fluorescence trace after normalization. Panel d) shows the deconvolved release trace (R(t) or release function), which is used in later analysis.

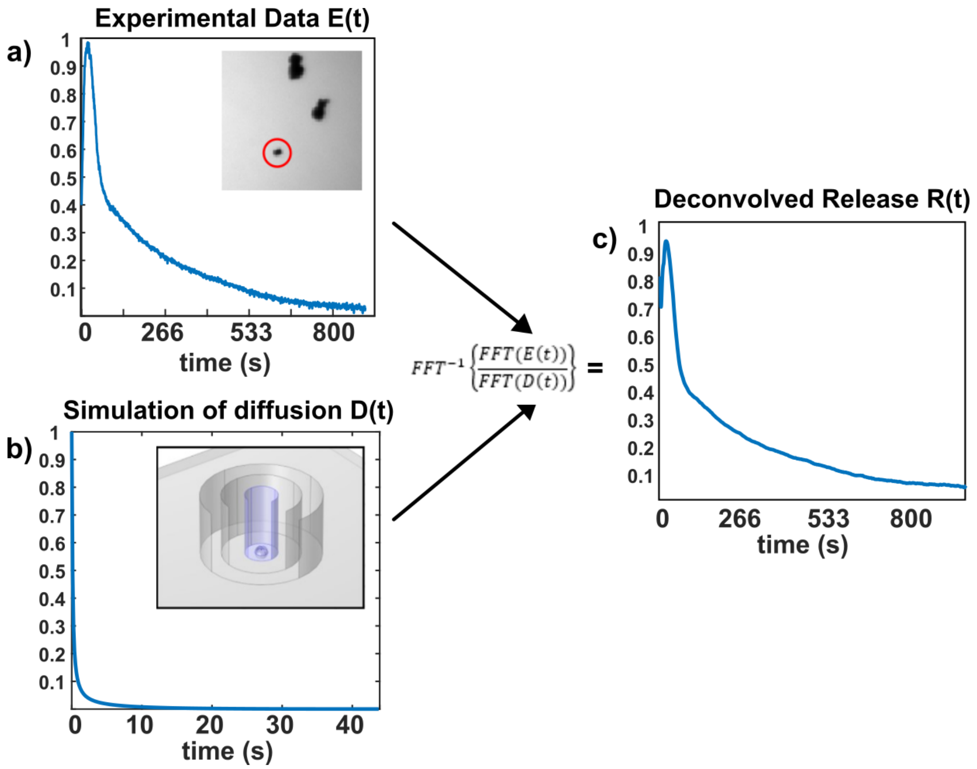

Figure S13. A closer look at deconvolution. a) Experimental trace E(t) (inset shows particle that gave this response, ROI circled in red) b) Simulated lithium diffusion D(t) (inset shows simulation geometry with cylindrical shells corresponding to each ROI. The ROI corresponding to the experimental data is highlighted in blue). The two are used to produce the deconvolved signal, c), the release function R(t).

As described in the main text and shown in Figure S15 below, various quantities can be used to describe the observed release traces. In the absence of a microscopically rigorous mechanistic model for lithium release, examination of these few scalar quantities was preferred over fitting or modeling the traces. This approach simplifies analysis and avoids issues of overfitting. For the processed traces, the intensities are in arbitrary units that result from the background subtraction, illumination correction, and deconvolution processes.

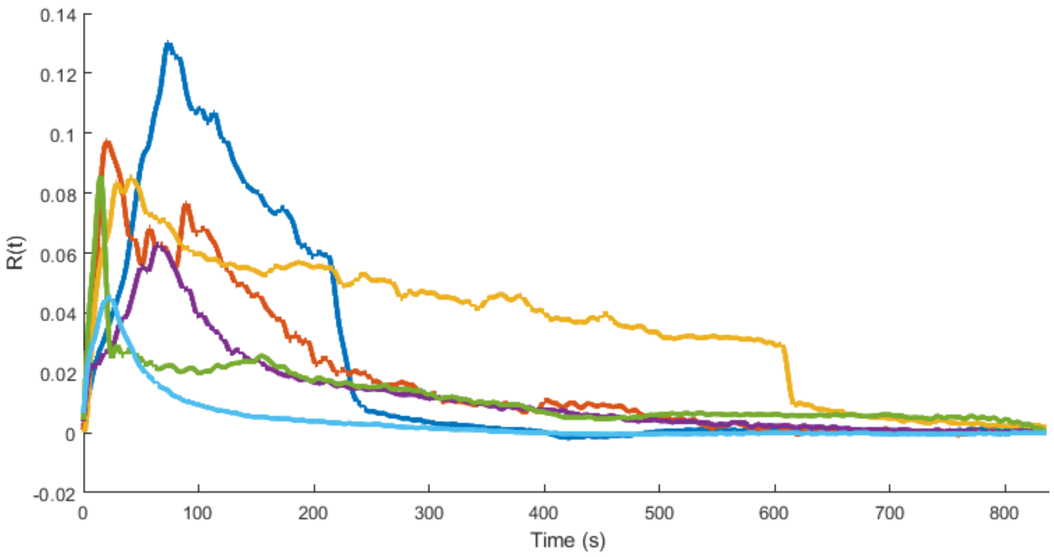

Figure S14. Release traces from the particles shown in main text Figure 3, overlaid on the same y-axis for direct comparison. A variety of release behaviors are observed, owing to heterogeneity of the samples.

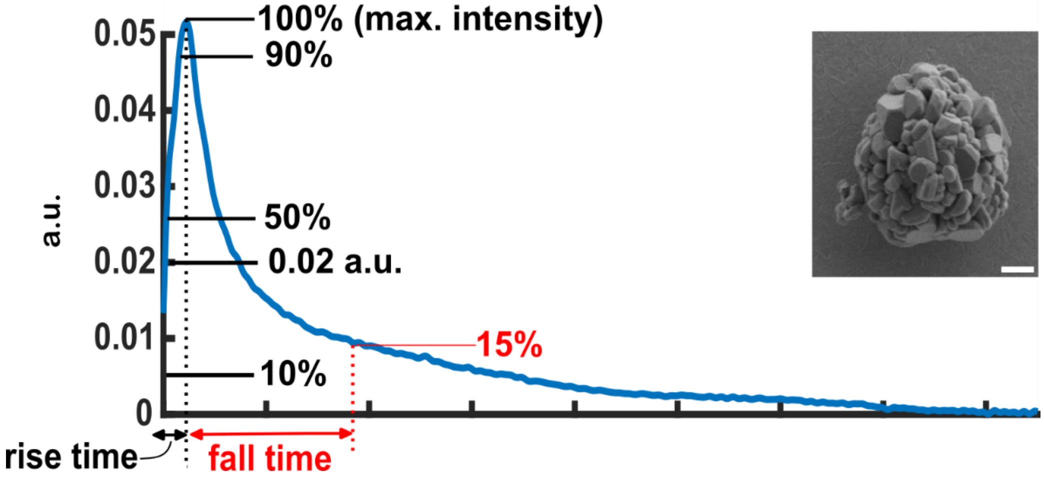

Figure S15. Trace from particle shown, with lines to highlight the quantities extracted from each.

The figure above shows a subset of the values extracted from the traces – many more are included in the analysis, but the figure has been simplified for clarity. Time to reach 10, 50, 90, and 100% of maximum intensity (black lines in the figure) are recorded, along with the intensities at that time. Additional features independent of rise time such as the time to fall from the max amplitude to 15% (in red) of maximum intensity were also extracted. 15% was chosen as a cutoff for fall time measurement (as opposed to a return to 0 amplitude) to account for experiments with lower signal/noise which may not return fully to the baseline. In addition to values relative to the peak, some values at absolute amplitude values were chosen for analysis, for example the time to reach 0.01 or 0.05 arbitrary units (a.u.) of intensity. In addition to these values shown on the graphs above, multiple intermediate intensity values,

rise times, and fall times are also extracted, as well as the integrated intensity over the entire trace. These values are stored in a database of particles, so as to be linked to the SEM data for later analysis (described below).

SEM Image Analysis

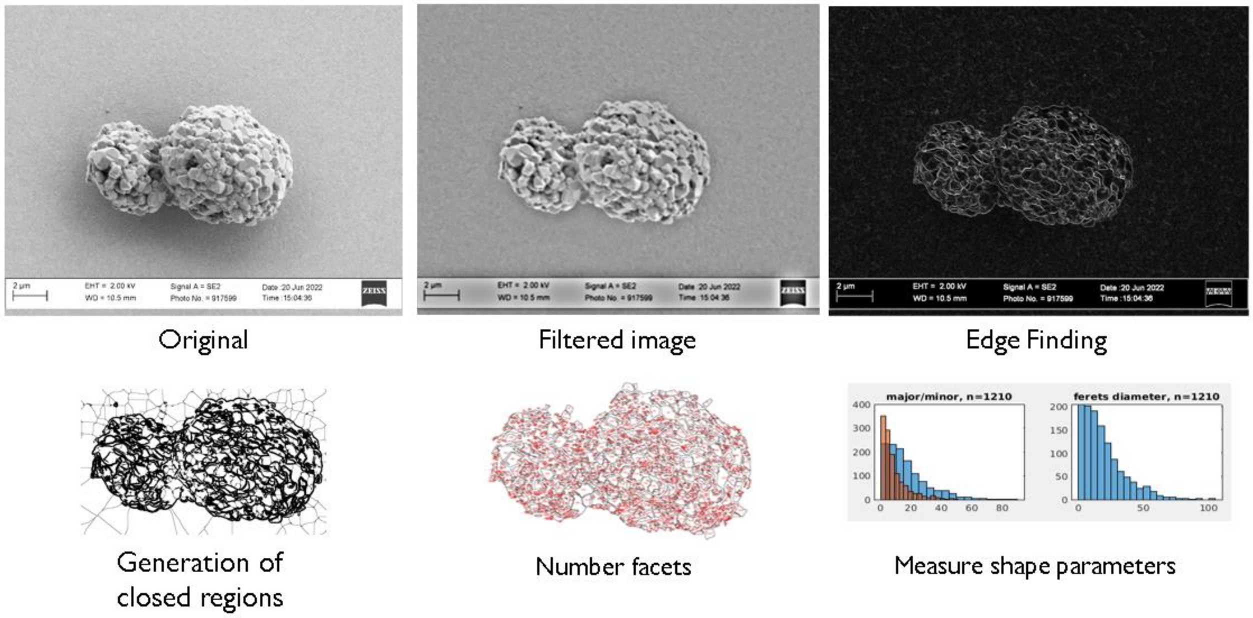

Figure S16. Example of SEM image analysis workflow.

SEM image analysis was performed with custom software written in MATLAB and ImageJ. A summary of this process is shown in Figure S16. As each SEM was taken with slightly different zoom level, the scales are not consistent. This issue is solved in an automated way by using an optical character recognition (OCR) protocol to read the text above the scalebar, along with some simple MATLAB image analysis to determine the scalebar length. The OCR model was trained on a subset of the SEM images and constrained to only recognize numbers and units that would be present in this dataset (the character set [0,1,2,u,n,m]). Any anomalous output would be presented to the user for confirmation. This scale value and length is fed to an instance of ImageJ running within MATLAB to set the scale for all subsequent measurements. After the scale is set, each SEM image is filtered with a bandpass and automatically cropped to isolate the particle and remove shadows. The filtered image is then passed through a variance filter, which replaces each pixel with a value corresponding to the neighborhood variance. This process highlights the edges of the facets within a particle. The variance image is thresholded automatically to generate a binary image, which is then passed to a watershed algorithm to ensure that the binary image is composed of closed regions. The resulting image can be analyzed with the built in Particle analysis function in ImageJ. This process was performed while selecting for regions of size 60-8000 pixels, in order to remove spurious small regions and to exclude any large regions outside of the particle, which can occasionally be generated by the watershed algorithm. The regions generated by this particle analysis can then be measured to determine the number of subparticles making up the particle being analyzed along with their individual areas, their diameters, and other measurements that describe their shape. The combination of all of these regions is used to determine the total area and

diameter of the particle being analyzed. All of these values are then stored in a database with the fluorescence information, when available, for each of the particles, along with tags defining whether a particle was found in both the SEM and fluorescence data sets, and whether it had a fluorescence response. The results of this analysis across all particles is summarized in the histograms below.

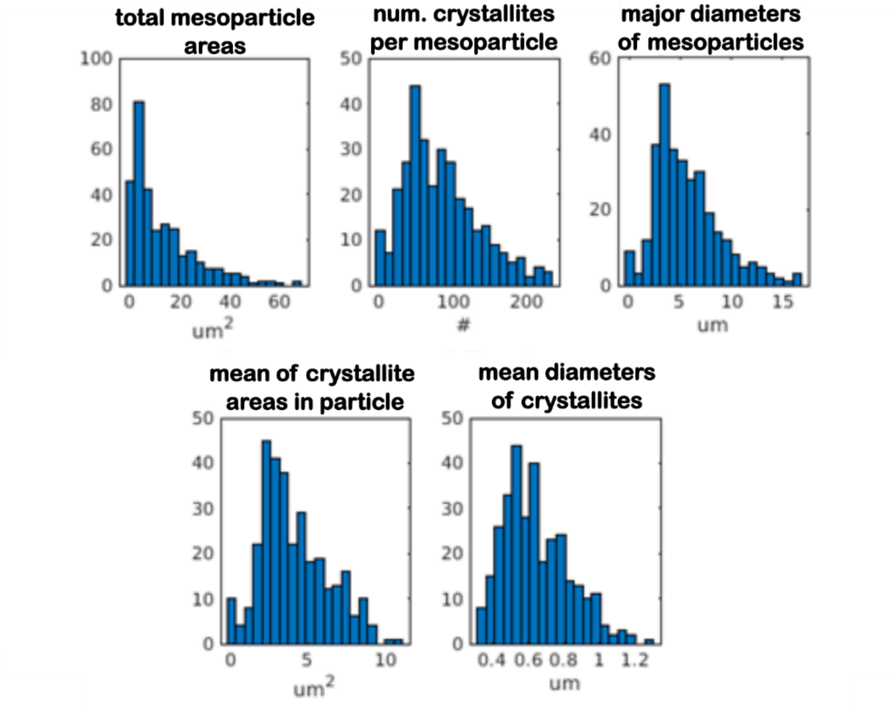

Figure S17. Histograms of various physical parameters describing the particles. These quantities, generated by the above analysis, show the wide range of particle sizes present.

Examination of Non-Responsive LCO Particles.

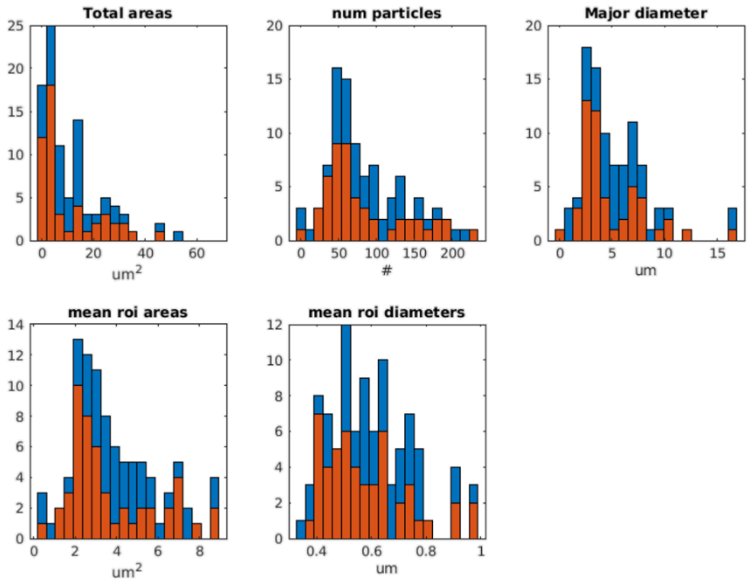

Figure S18. Subpopulation of non-responsive particles (red) shows no difference in distribution along the axes relating to area, number of constituent particles, or diameter when compared to the dataset as a whole (blue). Additionally, no significant difference is seen among the constituent crystallites' areas or diameters.

A significant portion of the particles analyzed after SEM were later observed to have no fluorescence response. This raises the possibility that there could be a common feature of these particles preventing their ability to release lithium or preventing good contact with the surface. If so, we would expect to see the non-responsive particles form a distinct subpopulation with particular physical parameters in the above SEM analysis. For example, one might expect that only the smallest particles, or the particles made up of the least crystallites to be non-responsive. The image analysis was repeated on all particles which did not show a response in experiments but had an SEM image available. As shown in Figure S18, none of the physical parameters measured on the SEM appeared to be characteristic of this subpopulation, indicating that there is nothing distinct about these particles' morphology causing the lack of response. While local variations in the ITO surface itself can account for differences in resistance, the commercially prepared ITO is relatively uniform and this does not seem to dominate the observed differences, especially given that particles in close proximity on the surface often had considerably different responses. A more likely dominant effect is differences in contact resistance between the LCO mesoparticle and the ITO surface arising from random variations in the particle orientations incurred during deposition, though the top-down SEM imaging used in this work does not allow us to probe this interface more closely. It is possible that these particles just had poor contact with the ITO surface, impairing their ability to respond to the applied voltage, and that the chances of experiencing that poor contact do not correlate to any structural parameter available in the SEM.

p-values and correlation coefficients (r) for data in Figure 5

p values

1.20E-05	6.78E-06	0.000827	1.09E-06	0.000317	0.001798	0.000488
0.531098	0.673909	0.438169	0.91695	0.767938	0.863515	0.430282
0.875155	0.828446	0.557668	0.563029	0.4178	0.82451	0.567726
0.658564	0.075298	0.733732	0.177346	0.584046	0.977146	0.730449
0.607643	0.043921	0.77191	0.172035	0.551028	0.966832	0.829211
0.402845	0.817573	0.319373	0.72689	0.792672	0.983389	0.440623

Correlation coefficients (R)

0.581108	0.594134	0.462221	0.632427	0.493279	0.434765	0.479643
-0.09165	-0.06165	-0.11332	-0.01529	0.04325	-0.0252	-0.11527
-0.02304	0.031769	-0.08582	0.084658	0.118398	0.032509	-0.08365
0.064733	0.256444	-0.04985	0.195894	0.080155	-0.0042	-0.05049
0.076005	0.29214	-0.04295	0.200397	0.088214	0.006164	-0.03197
-0.12354	-0.03419	-0.14681	-0.05174	0.03895	-0.00309	-0.11394

These tables (6 rows x 7 columns) correspond to the numerical values for p and r that are indicated by the color (r) and shading (p) in Figure 4 (6 rows x 7 columns) in the main text. Below is a 2d colorbar demonstrating the interplay of these two values, with the resulting color used for each ellipse in the correlation figures.

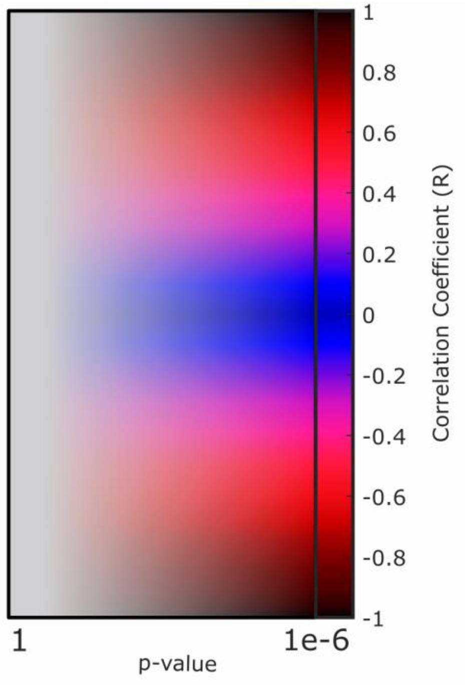

Larger Graph of Correlations between SEM Parameters and Release Function Traces

While specific correlations are highlighted in Figure 4 in the main text, a larger number of potential correlations between fluorescence data and SEM measurements are explored in **Figure S19**. Correlations among the various derived quantities are explored in Figure S20 and show many expected internal correlations (such as with peak intensity and integrated intensity). Figure S21 shows similar correlations for structural parameters derived solely from the SEM measurements (such as area and crystallite count).

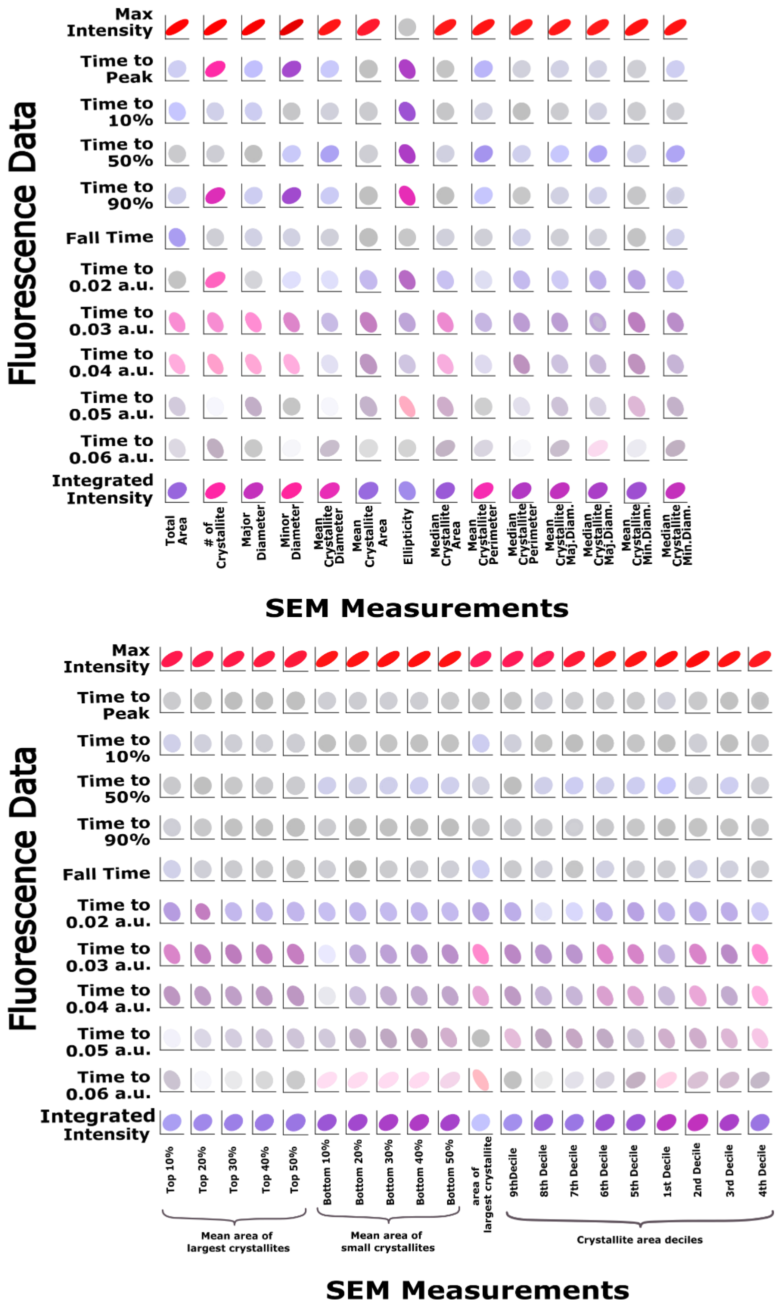

Figure S19. Scatter plots of each variable from the SEM and fluorescence analysis are made to explore the relationships between these parameters. Because these correlations are hard to interpret visually, a simple linear regression was calculated for each set and used to derive a correlation coefficient

and p-value for each set of variables. This figure represents each R and p value with an ellipse – Greater correlation coefficients are more red, and lower values more blue. Relationships with better p values are more saturated in color, and less significant p values are less saturated (closer to grey).

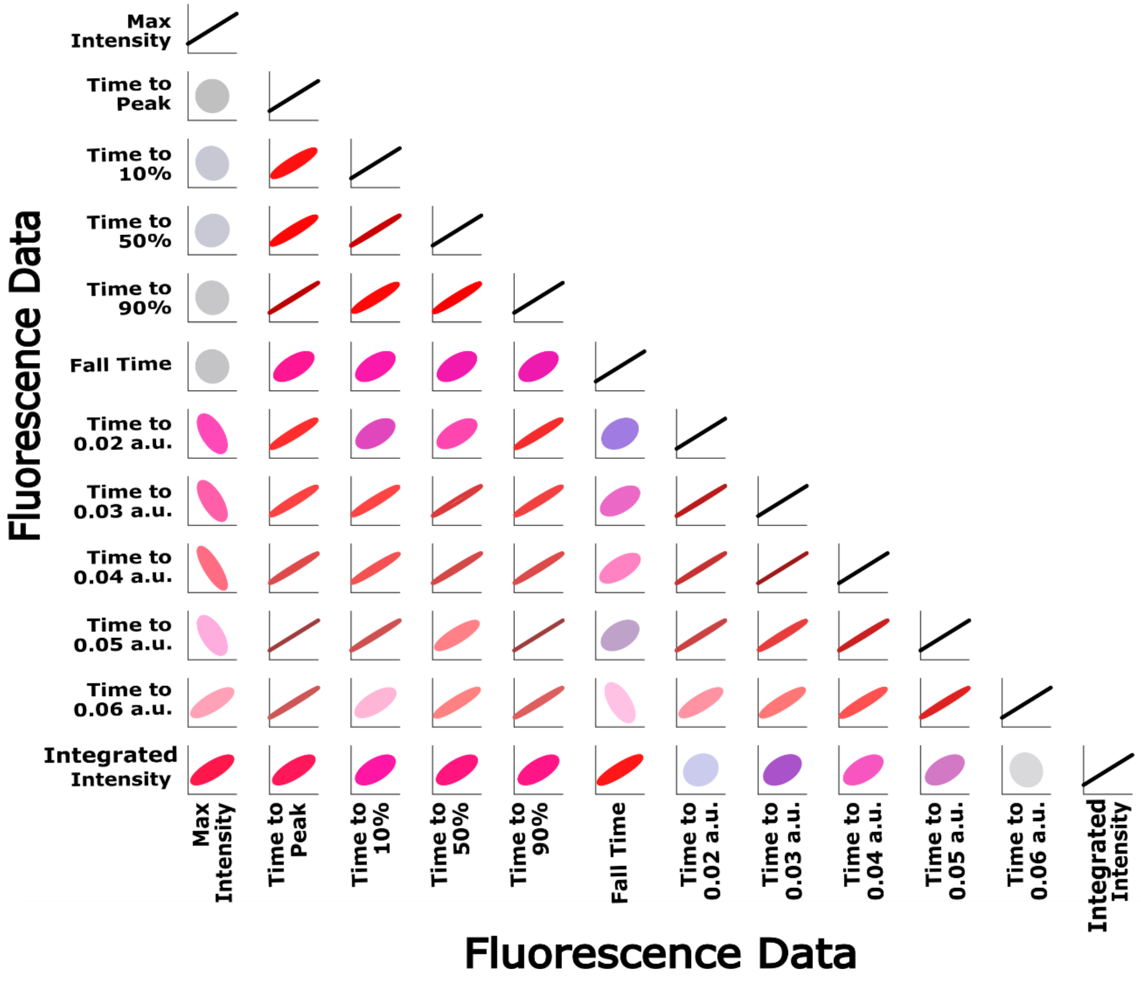

Figure S20. Representations of correlations between parameters derived from the deconvolved fluorescence traces (release functions).

Correlations between Morphological (SEM-derived) Parameters

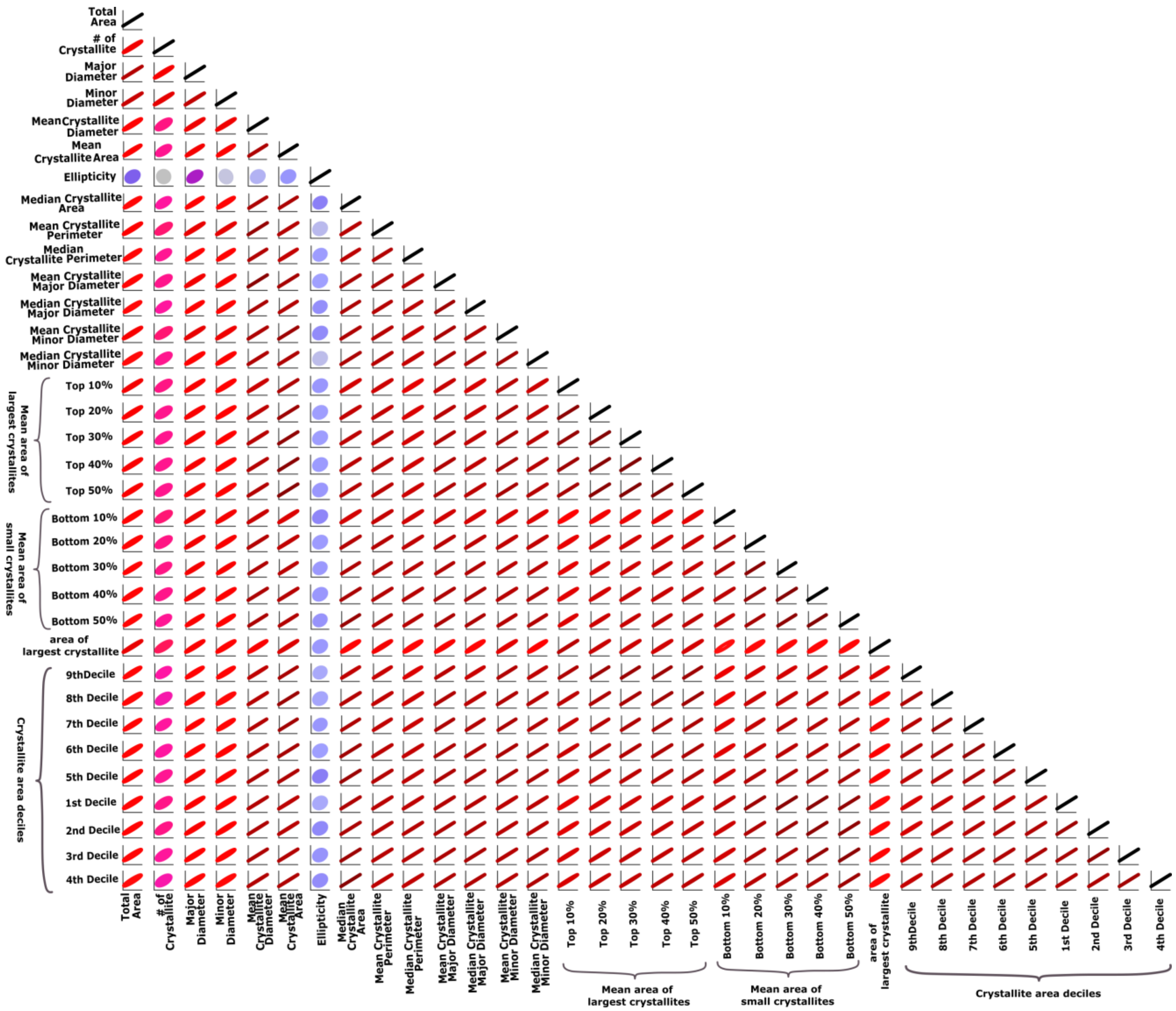

Figure S21. Correlations among SEM-derived parameters describing the physical shape of mesoparticles and their constituent crystallites. Mesoparticle size is seen to correlate with the number of crystallites as expected, and most of the parameters show at least some positive correlation.

Selection of Diffusion constant (D) value for COMSOL simulations

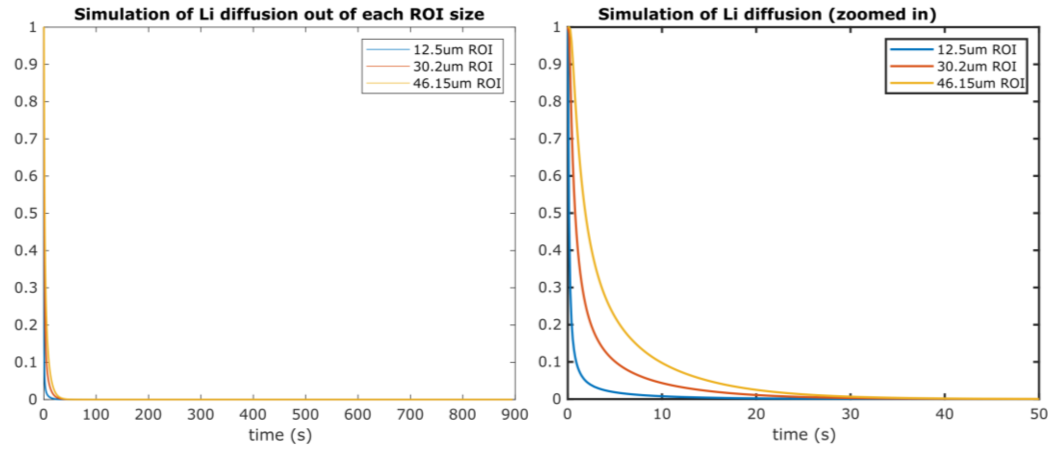

Figure S22. Graphs of concentration vs time obtained by simulation for each of the ROI diameters used. This graph is generated from simulations where D=1.00x10^{-10} m^2/s. Left: The full time series of ion concentration in each ROI (normalized to 1) shows the rapid diffusion of lithium ions out of the volume. Right: Zoomed in traces show differences between diffusion in each of the ROI sizes at early timepoints.

Using our own simulations of lithium-ion diffusion (details in main text), we can validate the selection of a D value as follows: We considered a range of potential D values (from 1.00x10^{-11} m^2/s to 5.00x10^{-10} m^2/s) as a starting point, based on literature precedent. For each potential D value, a diffusion simulation was performed, and multiple diffusion vs time functions, D(t), were generated corresponding to each of the region of interest (ROI) radii shown in Figure S23. The equivalent ROIs in the experimental data are then selected and processed as above to get the experimentally observed functions E(t) for each ROI.

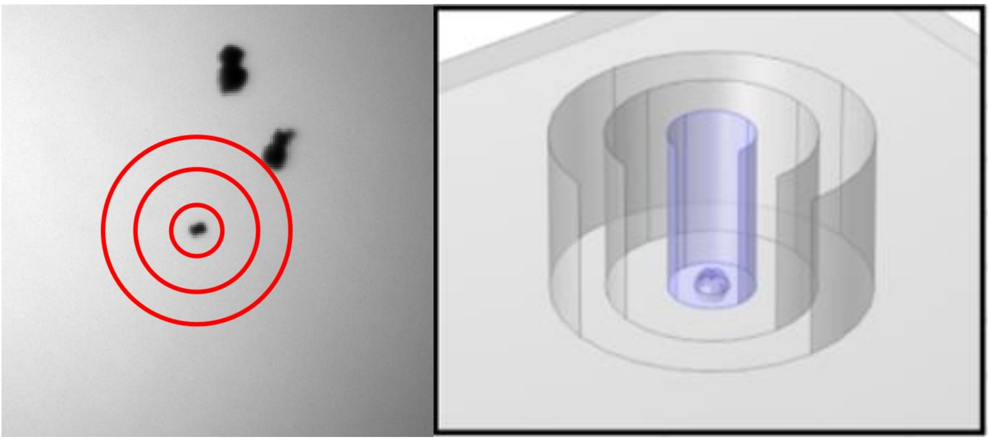

Figure S23. Example of the 3 ROI diameters around a single mesoparticle, left, and the corresponding geometry used in our simulations, right. All experimental traces in the main text use the innermost (12.5 um) ROI unless otherwise specified.

Each experimental E(t) function is deconvolved with the simulated diffusion functions of the same ROI diameter to generate a release function. If the selected diffusion constant accurately describes the diffusion in the experiment, then the different release functions resulting from different ROI radii should be substantially similar (because the release function should be the same regardless of ROI radius once the diffusion component is removed). On the other hand, an incorrect D value will cause the release traces to diverge from one another. Since only one release function of lithium should describe the experimental data, this inconsistency rules out those incorrect D values. Simulations with varying D (from 1.00×10^{-11} m^2/s to 5.00×10^{-10} m^2/s) were performed. An error value is calculated to quantify the difference between these curves for each case. The D value which gave the smallest error among the 3 calculated release functions was chosen as the optimal diffusion constant. This procedure was repeated for multiple experimental datasets to confirm the chosen value. In our experiment the optimal value was determined to be $D=1.0 \times 10^{-10}$ m^2/s, which is within the range observed in the literature. Example graphs of this analysis are shown below.

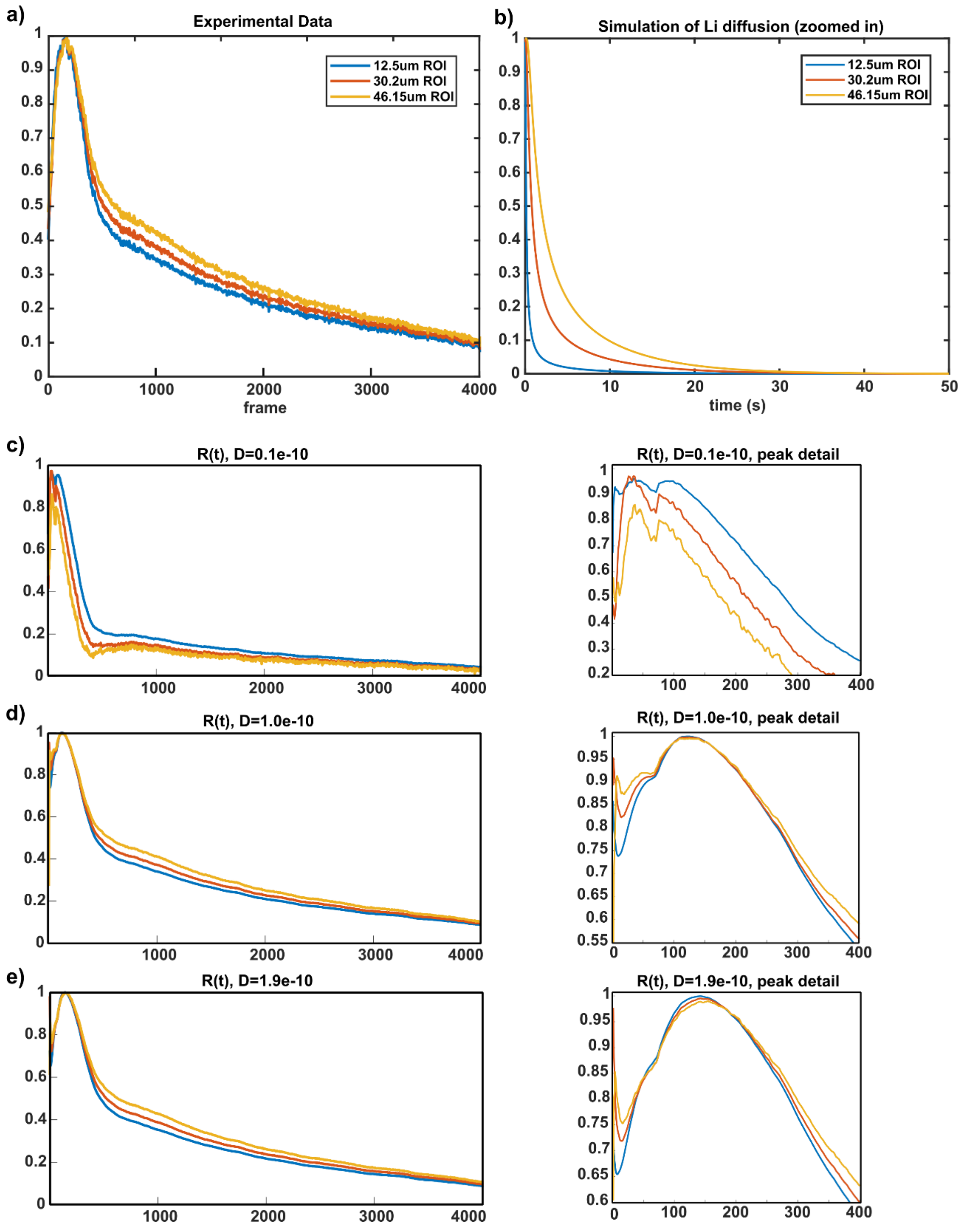

Figure S24. Example comparing deconvolution of traces derived from different ROI sizes using 3 values of D (0.1 x10^{-10} m^2/s, 1 x10^{-10} m^2/s, and 1.9x10^{-10} m^2/s). a) Experimental data, showing traces from all 3 ROI sizes. b) Example simulated D(t) traces for D=1 x10^{-10} m^2/s. Note that each deconvolution here uses a different simulation corresponding to its chosen value of D. c) Release traces produced when using D=0.1 x10^{-10} m^2/s. Graph on right shows a zoomed in detail of the peak area, showing poor agreement. d) Release traces produced when using D=1 x10^{-10} m^2/s. Graph on right shows a zoomed in detail of the peak area, showing much better agreement. e) Release traces produced when using D=1.9

x10^{-10} m^2/s. Graph on right shows a zoomed in detail of the peak area. A slightly poorer alignment of the release traces is seen at the peak, as compared to D=1 x10^{-10} m^2/s.

Control experiments with no LCO particles

To confirm that the response seen when applying an oxidizing voltage is only due to the release of lithium ions from particles of LCO, experiments were conducted on electrodes that were prepared as shown above, but without LCO particles present. No increase in fluorescence intensity is observed in this case. Interestingly, an increase is seen when applying increasingly reductive potentials (such as during a CV), but these potentials are never applied during monitoring of lithium release from LCO. A key need for performing the background subtraction described above is that HPNO is observed to photobleach in a manner that is consistent. Notably the bleaching rate is dependent on applied voltage, as shown in Figure S26 below. CVs of HPNO solution (without lithium or LCO present) display an irreversible oxidation peak at ~0.35 V, shown below in Figure S27.

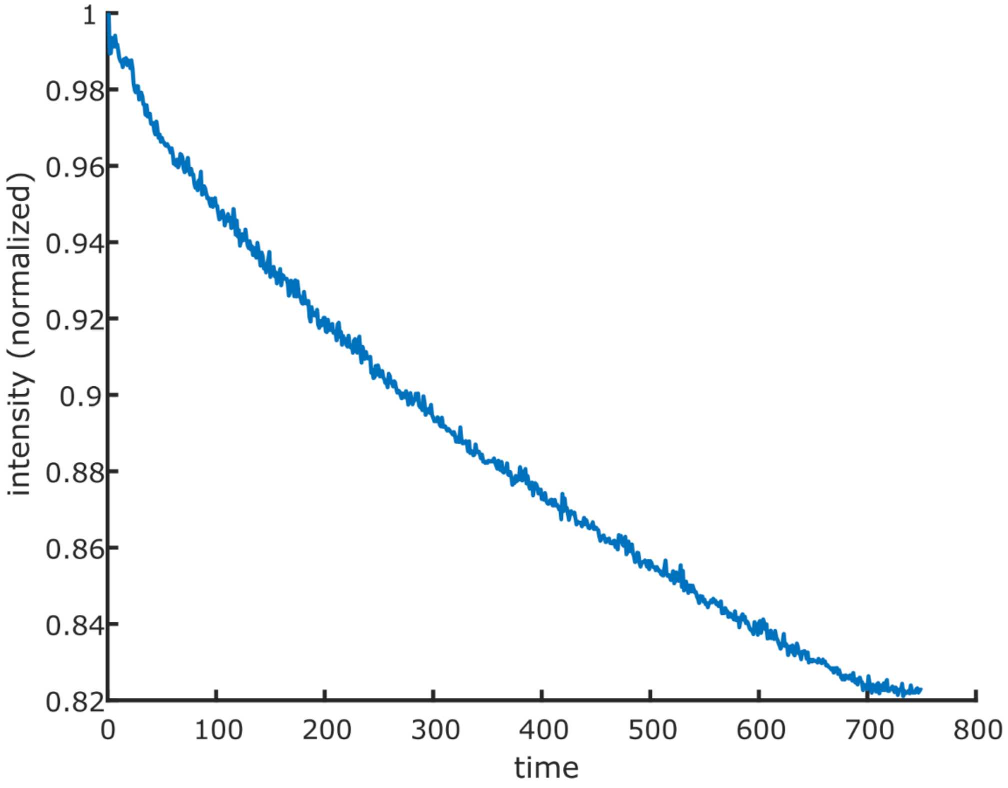

Figure S25. Fluorescence trace taken from an experiment run on a coverslip with no LCO. The HPNO bleaches over time, lowering the intensity observed. No turn-on response is seen without the presence of Li ions.

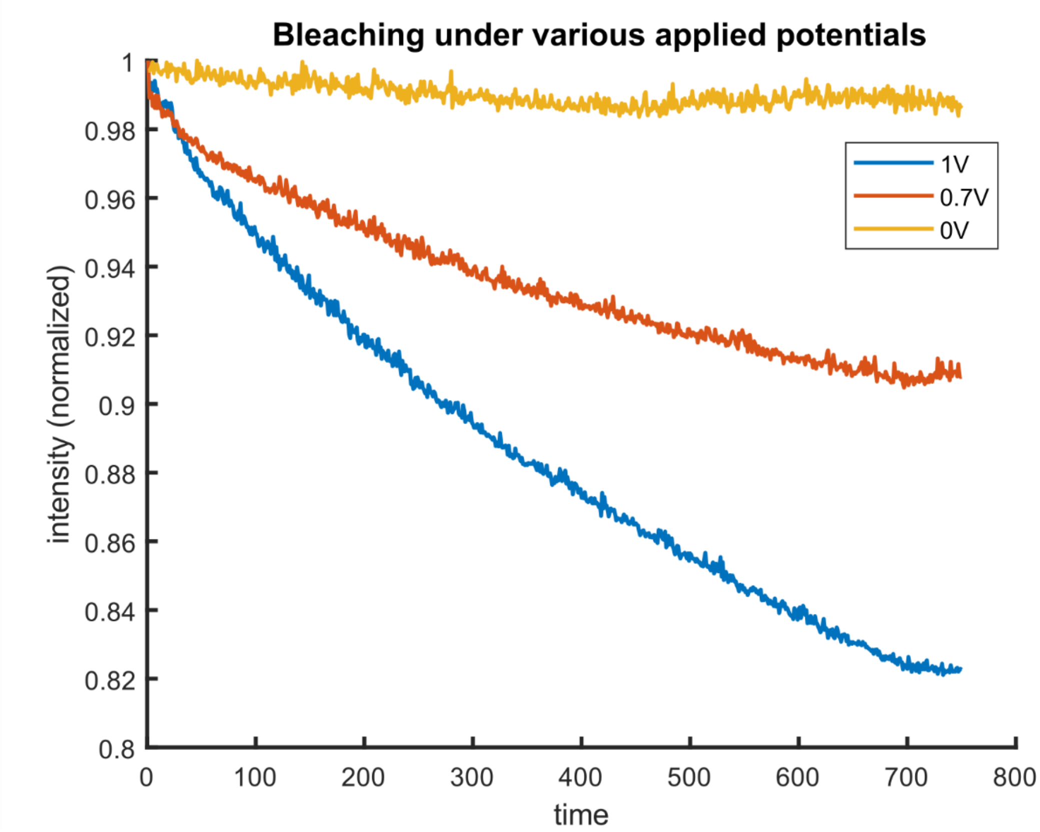

Figure S26. Photobleaching controls under various applied potentials. Photobleaching of HPNO is slow at 0V under experimental illumination conditions. Application of oxidizing voltages accelerates HPNO degradation and apparent photobleaching.

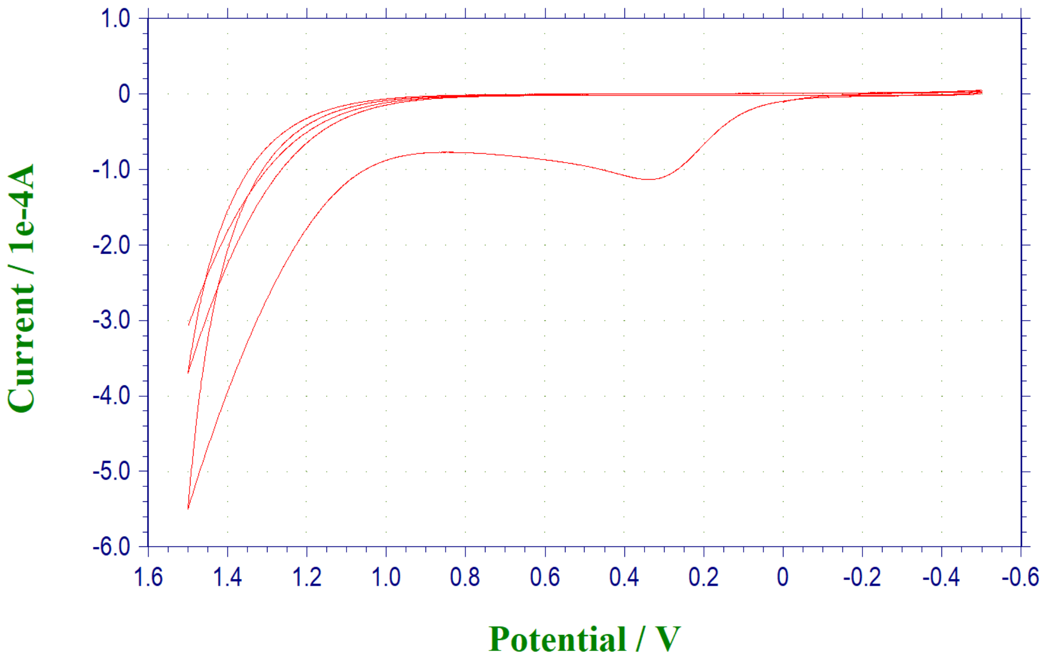

Figure S27. Cyclic voltammogram of HPNO dye solution in our cell geometry (no LCO present), 3 cycles. The first cycle shows an irreversible oxidation peak at ~0.35V.

ToF-SIMS Mapping of Lithium Ion Distribution

To provide additional support that fluorescence increase was due to lithium ion release, we obtained independent evidence that LCO particles were releasing lithium ions. Time-of-flight secondary ionization mass spectrometry (ToF-SIMS) was used to map the spatial distribution of lithium ions before and after discharge. In these experiments, LCO particles were prepared at higher concentrations where larger clumps of particles were observed. Discharge was triggered by fifteen 10-second oxidation cycles at 1V. ToF-SIMS experiments were operated in positive ion imaging mode with charge compensation. Significant transfer of lithium ions to the immediate vicinity of LCO particle clumps were observed, as expected, corroborating the fluorescence microscopy results.

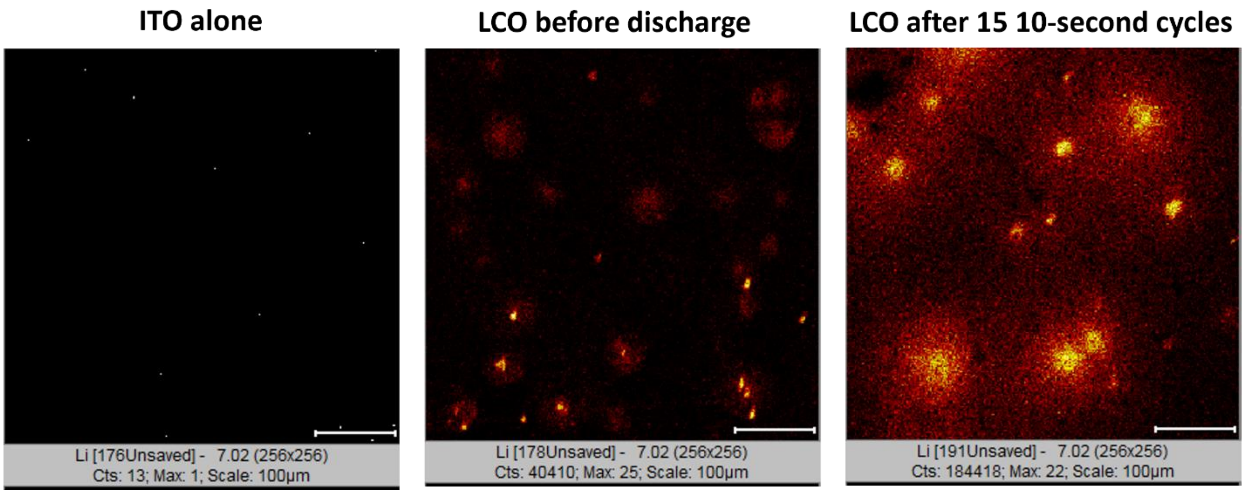

Figure S28. ToF-SIMS imaging of lithium ion spatial distributions.

Cyclic Voltammogram of LCO particles

Throughout this work a silver wire pseudoreference electrode is used (as described in the electrochemical methods). This reference was chosen due to its chemical compatibility and relative air stability, along with considerations for the sample and imaging geometry used. All fluorescence traces in this work are collected while under 1V applied potential relative to this pseudoreference (unless otherwise noted), a value which was chosen to maximize observed fluorescence signal while minimizing oxidation of solvent and dye solution.

In order to understand where this 1V applied potential lies relative to the $LiCoO_2$ couple, cyclic voltammograms were taken on a coverslip coated with LCO particles. The particle coverage was much greater than would be seen in a typical fluorescence experiment in order to obtain a measurable current response. An irreversible oxidation process is observed at 1.8V vs our pseudoreference electrode. LCO is known to oxidize at approximately 4.1-4.2V vs Li/Li^+,[2-4] though this number is dependent on electrolyte composition and concentration. Therefore, the 1V vs the pseudo reference is approximately equal to 3.3-3.4V vs Li/Li^+.

LCO-based cells examined in the literature are often characterized by galvanostatic charging/discharging. The voltage at which the majority of the LCO charging process takes place is approximately 3.9 V vs Li/Li+, depending somewhat on the electrolyte composition. While this voltage is still greater than the applied voltage in this work, our experiments examine the crucial initial Li^+ release from the LCO particle that occurs at the beginning of a charge cycle, which typically occurs at lower potentials.

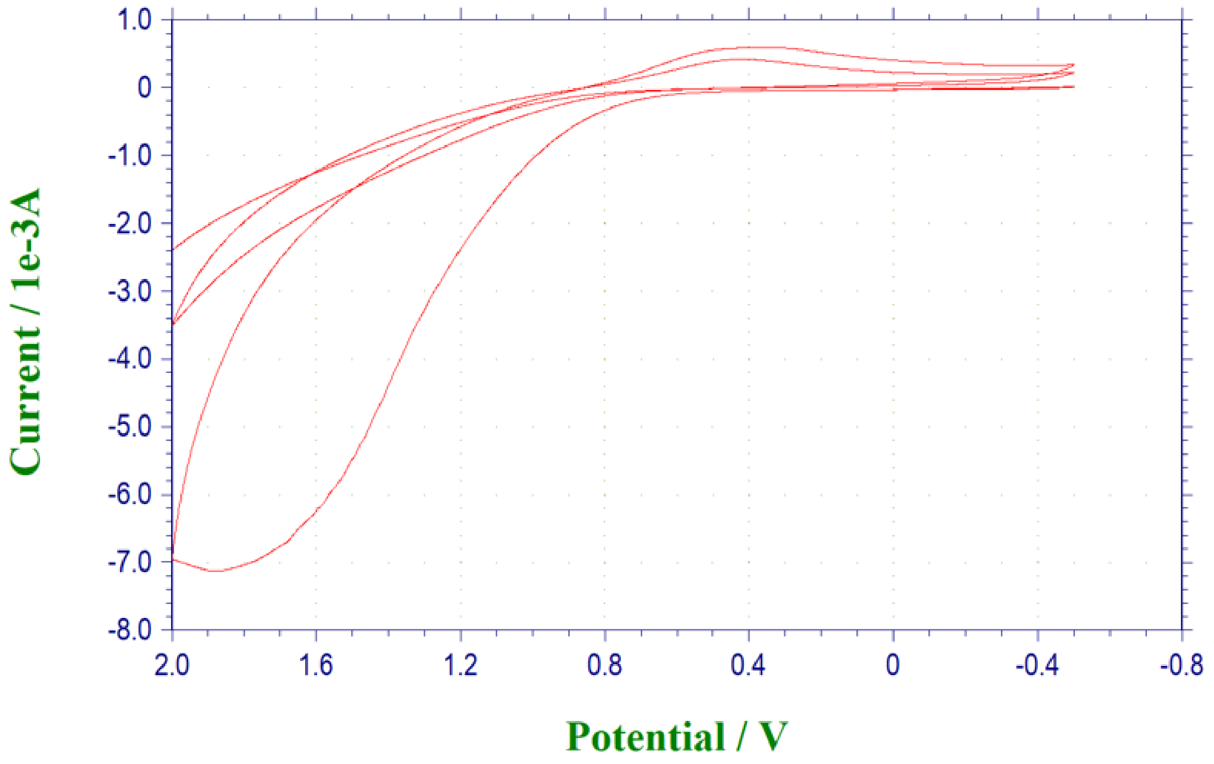

Figure S29. CV of heavily LCO coated ITO coverslip. Potential was scanned from 0V to 2.0 V, then back to -0.5V. The scan rate was 100mv/s. The oxidation appears mostly irreversible after lithium ions are released into the bulk of the surrounding solution, as expected due to the lack of Li^+ in the sample electrolyte.

HPNO-free Control Experiment

To ensure that all fluorescence observed during the experiments originated from HPNO-bound lithium ions we ran a control experiment in an HPNO-free electrolyte solution. The HPNO-free electrolyte solution is composed of $TBAPF_6$ (0.1 M) and TEA (4% v/v) in propylene carbonate. Upon application of 1V over a span of a minute no change in fluorescence intensity around the LCO particles was detected.

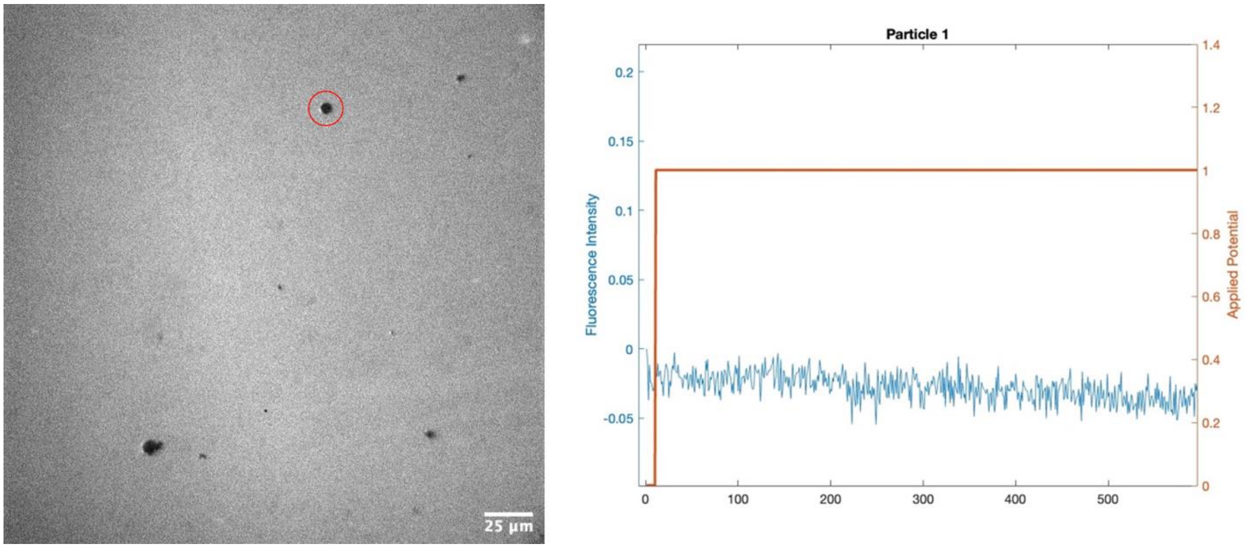

Figure S30. Intensity trace of LCO particle after 1V application in HPNO-free electrolyte solution. No change in fluorescence is detected.

Fluorescence-Concentration Calibration

To directly measure the concentration of lithium ions, present after release, we switched to a Nikon Ti2 Yokogawa W1 spinning disk scanning confocal microscope (405 nm ex, Plan Fluor 40x oil obj, NA=1.30, Ziva CSU 445/49 BP filter). LCO particles were exposed to 1.5V over a 5-minute period to track Li release. Confocal imaging allowed for a direct measurement of the excitation volume so fluorescence intensities could be directed correlated to a Li ion concentration with a LiBr calibration curve (Figure S31). From here, we selected pixels that fell within the established calibration range and calculated the average lithium concentration per frame across all pixels in range. Based on calibration slides with the same objective magnification we knew our pixel width was approximately 546 nm. For the pixel depth we used Nikon's resolution calculator for spinning disk microscopy which estimated an axial height of 781 nm using our optical parameters. These dimensions provided a pixel volume of $2.3x10^{-19}$ L which allowed us to convert from average lithium concentration across a given selection to a molar quantity of lithium. Based on previous diffusion measurements[1] we estimated that each frame (200 ms exposure) will contain a fresh batch of lithium ions as lithium will diffuse, on average, 15.9 microns each frame and quickly move out of focus.

To calculate the total amount of Li released from a single LCO particle the molar release curve was integrated over the entire release time. The total amount of lithium released ranged from $1.37x10^{-17}$ - $3.35x10^{-17}$ moles depending on the size of the mesoparticle analyzed. Once the quantity of Li released is

known, the total charge released from a single particle can also be calculated assuming that each Li ion has a +1 charge. This gives a value of 3.22×10^{-12} coulombs released in total for our largest release event.

To measure the proportion of lithium released from LCO particles we first estimated the size of each particle by counting the pixel area occupied by the particle and multiplying by the axial height (781 nm) of the confocal system. The total mass of each particle was estimated by multiplying the particle's volume by its provided tap density (2.6 g/mL). The number of moles of lithium per particle was then found using the assumption that each particle is 7.02% Li by mass. From here the total number of moles released is compared to the estimated total Li content to find the proportion of the lithium that was displaced from the particle. We found that the particles released between 0.55% - 1.11% of their total Li content.

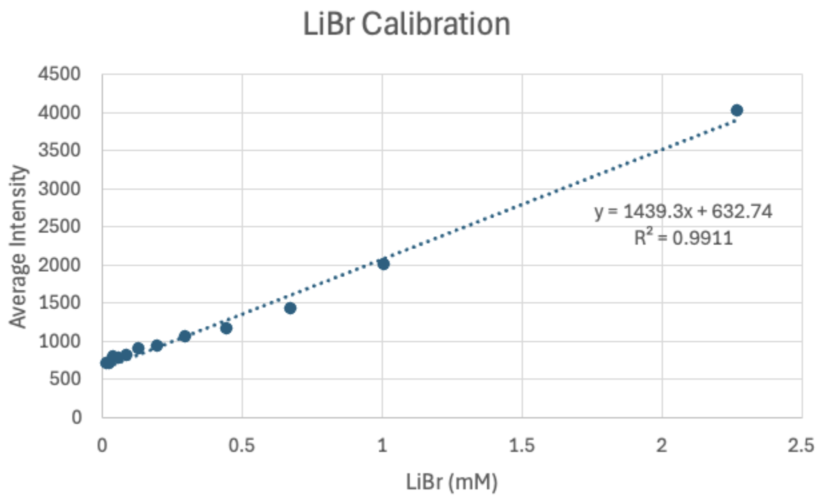

Figure S31. LiBr calibration curve from confocal microscope with fit: Intensity = 1439.3[LiBr]+ 632.74, R^2 = 0.9911.

Uneven Lithium Release

Confocal microscopy also allows for spatial monitoring of Li concentration. We’ve observed multi-domain mesoparticles release lithium non-simultaneously with millisecond delays between release events across different regions of the particle which highlights the diversity of dynamics even within a single particle.

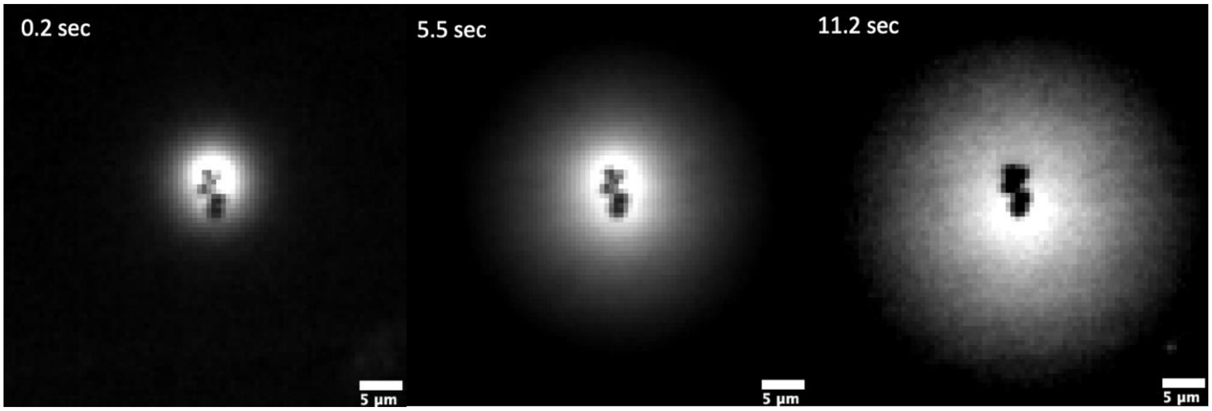

Figure S32. Non-even lithium release from an example LCO particle with delayed lithium release in the bottom domain compared to the top. The bottom node continues to release lithium even once the top has been depleted of available lithium.

Galvanostatic Conditions

Lithium release experiments were also run under galvanostatic conditions (I = 0.1545 mA) to see if lithium release rate could be controlled at specific currents. We observed that the LCO particles successfully release lithium under these conditions, and the timing of release was dependent on the potential step.

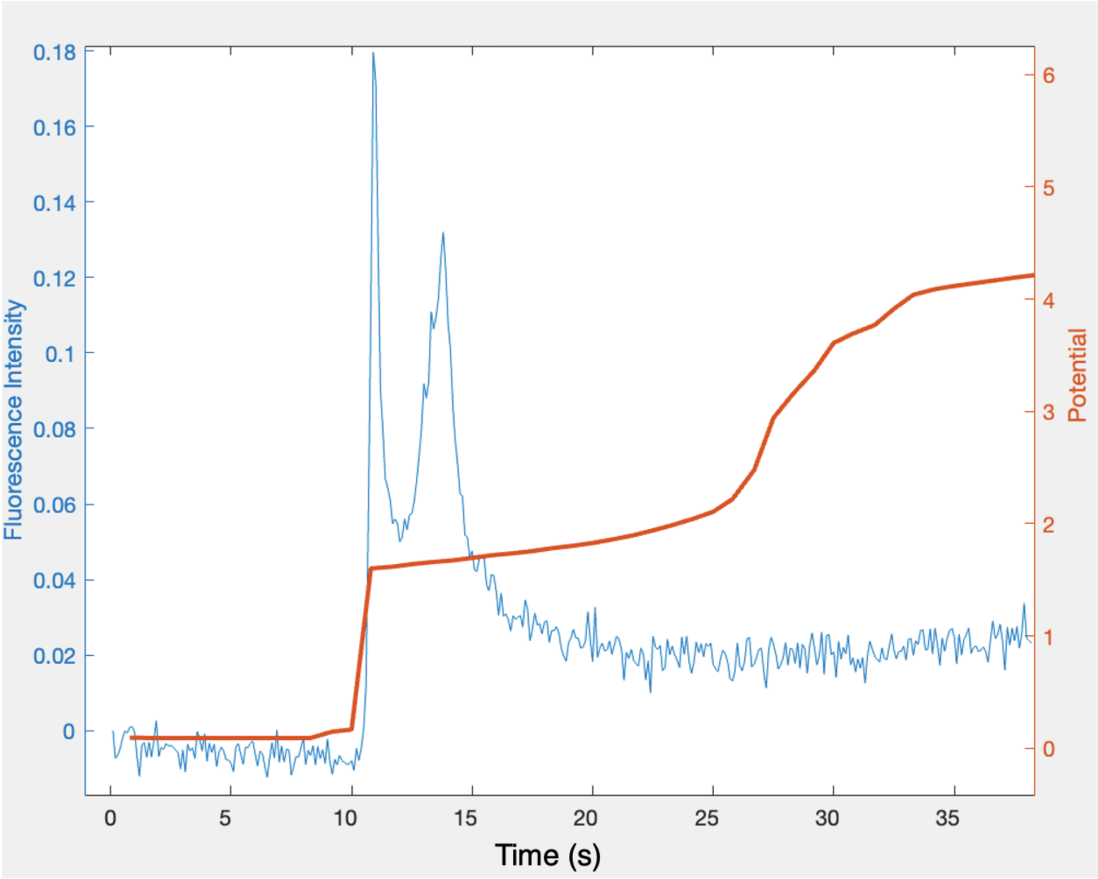

Figure S33. Intensity trace from LCO particle after applying a constant 0.1545 mA current with a 10 second delay to the ITO coverslip. Fluorescence is observed upon initial potential step needed to apply specific currents.

(1) Padilla, N. A.; Rea, M. T.; Foy, M.; Upadhyay, S. P.; Desrochers, K. A.; Derus, T.; Knapper, K. A.; Hunter, N. H.; Wood, S.; Hinton, D. A.; et al. Tracking Lithium Ions via Widefield Fluorescence Microscopy for Battery Diagnostics. *ACS Sens* **2017**, *2* (7), 903-908. DOI: 10.1021/acssensors.7b00087.
(2) Liu, J.; Liu, N.; Liu, D.; Bai, Y.; Shi, L.; Wang, Z.; Chen, L.; Hennige, V.; Schuch, A. Improving the performances of LiCoO2 cathode materials by soaking nano-alumina in commercial electrolyte. *Journal of the Electrochemical Society* **2006**, *154* (1), A55.
(3) Qian, J.; Liu, L.; Yang, J.; Li, S.; Wang, X.; Zhuang, H. L.; Lu, Y. Electrochemical surface passivation of LiCoO2 particles at ultrahigh voltage and its applications in lithium-based batteries. *Nature communications* **2018**, *9* (1), 4918.
(4) Wu, S.; Lin, Y.; Xing, L.; Sun, G.; Zhou, H.; Xu, K.; Fan, W.; Yu, L.; Li, W. Stabilizing LiCoO2/graphite at high voltages with an electrolyte additive. *ACS applied materials & interfaces* **2019**, *11* (19), 17940-17951.
